# Characterization of flavor profile of Steamed beef with rice flour using gas chromatography-ion mobility spectrometry combined with intelligent sensory (Electronic nose and tongue)

**DOI:** 10.3389/fnut.2024.1435364

**Published:** 2024-08-20

**Authors:** Tianyang Wang, Lian Yang, Yiling Xiong, Baozhu Wu, Yang Liu, Mingfeng Qiao, Chenglin Zhu, Huachang Wu, Jing Deng, Ju Guan

**Affiliations:** ^1^Cuisine Science Key Laboratory of Sichuan Province, Sichuan Tourism University, Chengdu, Sichuan, China; ^2^College of Food and Biological Engineering, Chengdu University, Chengdu, Sichuan, China; ^3^College of Food Science and Technology, Southwest Minzu University, Chengdu, Sichuan, China

**Keywords:** Steamed beef with rice flour, intelligent senses, GC-IMS, free amino acid, chemometrics

## Abstract

The intelligent senses (Electronic nose and tongue), were combined with headspace gas chromatography-ion mobility spectrometry (HS-GC-IMS) and free amino acid were used in combination to determine the aroma and taste components during the processing of Chinese traditional dish Steamed beef with rice flour (SBD). The findings revealed that E-nose and E-tongue, could clearly distinguish and identify the aroma and taste of SBD. A total of 66 volatile substances and 19 free amino acids were identified by HS-GC-IMS and amino acid analyzer, respectively. The highest contribution to aroma in the production of SBD was alcohols, esters and aldehydes. Further analysis of relative odor activity showed that 3-Methylbutanol-D, 3-Methylbutanol-M and 3-Methylthio propanal is the marinating stage (T2) main aroma components. Ethyl 3-methylbutanoate-M and Ethyl 3-methylbutanoate-D were the main aroma components in the seasoning stage (T3). Additionally, the calculation of the taste activity value showed that Glutamic contributed significantly to the umami of SBD. Alanine was a representative taste component in the marinating stage (T2), while Proline, Aspartic, Lysine, Glutamic, Valine, Arginine, and Histidine were characteristic amino acids of the seasoning stage (T3). Consequently, this study offers valuable insights into the industrial-scale production and flavor regulation of SBD products.

## 1 Introduction

With the rapid pace of modern life, the demand for prepared dishes in the food market has been growing steadily (1, 2). The production of classic folk dishes using industrial technology is an important way to develop newly prepared dishes and improve production efficiency. However, there is no specific standard for the aroma characteristics of folk classic dishes, which mainly depends on the chef’s experience, and it is challenging to maintain the uniqueness of the aroma characteristics of the dishes in industrial production (3, 4). Therefore, exploring the characteristic flavor of classic dishes, which can provide a specific, quantifiable description of the flavor, is a necessary means of standard industrial production (5, 6).

**Figure 1:**
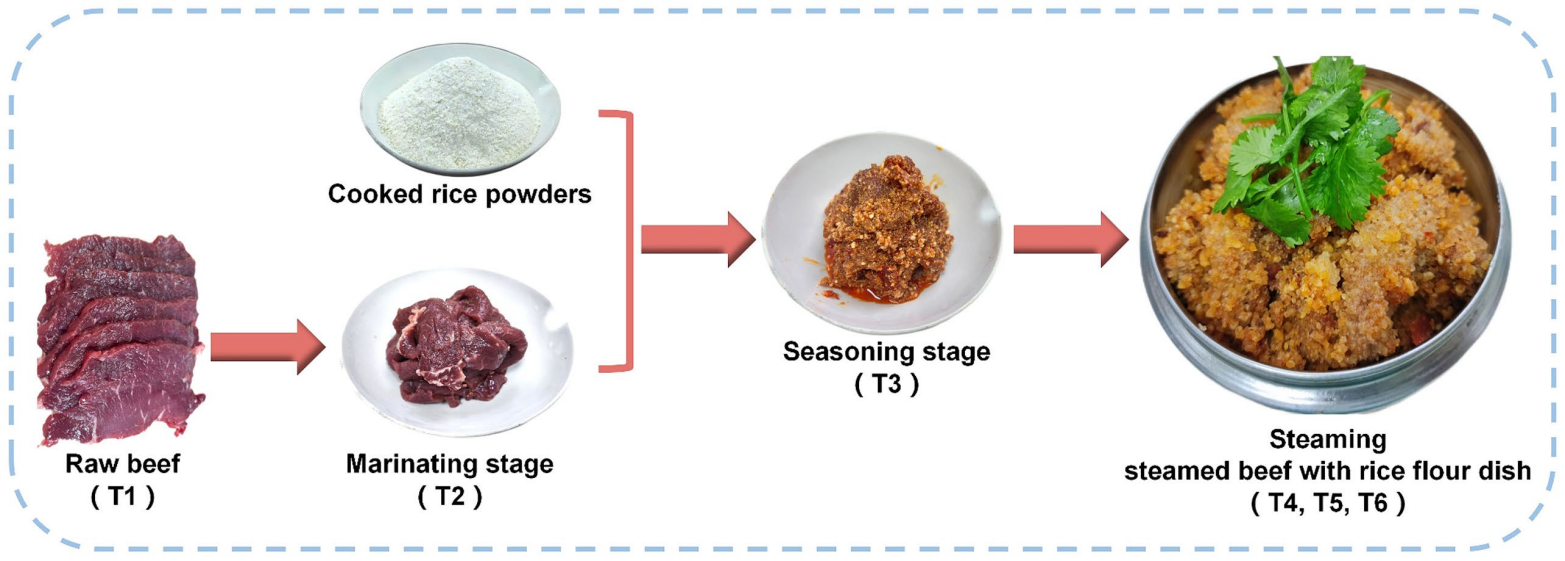
Schematic diagram of the different processing of Steamed beef with rice flour dish (SBD).

Steamed beef with rice flour dish (SBD) is a classic dish of Sichuan cuisine, the first of China’s eight major cuisines, and its origins can be traced back to the Qing Guangxu Dynasty (1862 AD). The unique and complicated process involved in the production of SBD is the result of a long history. The recipe for this dish includes beef and cooked rice flour, which, together with its specific cooking methods, endow SBD with its superb flavor and taste. Its critical processing consists of two main stages: marinating and steaming (Figure 1). In particular, adding rice flour makes the beef taste even smoother and adds layers of texture to the meat. On the other hand, SBD has long been well-known by many domestic consumers and is widely recognized in overseas Chinese communities and internationally. However, there are no studies on the characteristics and changes of aroma formation and taste characteristics of SBD during the cooking process. This limitation hinders the effective monitoring and further improvement of the industrialized SBD product quality.

Food flavor is one of the most essential characteristics of overall palatability and quality and is an important driver of consumer preference (7). In addition to the complexity of the dish’s make a profile, dishes’ aroma and taste profiles could not be obtained by a single assay (4). Together, GC-IMS, E-nose, and E-tongue were applied effectively to analyze the aroma and taste substances of food matrices such as meat products, aquatic products, tea, and fruit wine (8–11). Compared to gas chromatography-mass spectrometer (GC–MS), gas chromatography-ion mobility spectrometry (GC-IMS) can provide a more intuitive representation of the characteristics and differences in volatile compounds among samples (12). Meanwhile, HS-GC-IMS can directly detect SBD through headspace vials without the need for enrichment and concentration, greatly enhancing the efficiency of detecting aroma components in SBD. To date, the use of intelligent sensory techniques in combination with advanced instrumentation for the study of flavor profiles of food products has become a meaningful way to produce classic folk dishes by industrial means and to develop new pre-prepared dishes and food products (13). It provides more comprehensive, robust, and objective scientific information (14). Previous studies have successfully used intelligent sensory techniques to explore the flavor characteristics of classic folk dishes during the cooking process, such as Dongpo pork, Tomato sour soup beef, and Dezhou braised chicken (5, 8, 15).

This study investigated the labeling and formation of characteristic aroma and taste substances in the SBD during processing using intelligent sensory combined with HS-GC-IMS and amino acid analysis techniques and combined relative odor activity value (ROAV) and taste activity value (TAV) calculations. This study will help provide data on the formation of aroma and flavor at different stages of the production process of this classic dish and provide a theoretical basis for the industrial production and development of SBD.

## 2 Materials and methods

### 2.1 SBD cooking model and sample preparation

The SBD cooking model of this study was prepared according to the local standard “Technical Specification for Chinese Sichuan classic dishes (DB51/T 1728-2014).” According to the request, the fresh tenderloin from the adult yellow beef is selected for preparation (Food Technology Company, Chengdu, China). Fresh raw beef (T1) was first cut into slices (10 × 1 × 5 cm) and marinated with ginger, cooking wine and salt for 10 min (T2). Then add cooked rice noodles, Pixian pea sauce (JuanCheng, Chengdu, China) and condiments for seasoning (T3) in a steam pot for 20 min (T4), 40 min (T5), and 60 min (T6). This cooking model was repeated three times (Figure 1).

### 2.2 E-nose analysis

The electronic nose (FOX 4000, A MOS, France) is equipped with 18 sensor chambers that can identify classes of volatile compounds. The sensor’s performance in recognizing aromas is shown as follows: PA/2, P30/1, P30/2, TA/2, and LY2/AA were sensitive to organic compounds, LY2/LG, P40/1, P40/2, T40/2 and T40/1 were sensitive to gases with high oxidizing power, LY2/G and LY2/gCTI were sensitive to amines; LY2/Gh is selective for anilines, LY2/gCT is sensitive to alkanes and aromatic components, T30/1 is sensitive to polar compounds, P10/1 is sensitive to non-polar compounds; P10/2 is sensitive to alkanes, T70/2 is sensitive to aromatic compounds (16, 17).

Accurately weighed 2 g of crushed sample, capped with polyethene sealing cap membrane and placed into a 10 mL headspace vial for electronic nose, and incubated and equilibrated for 5 min at an incubation temperature of 40°C before injecting 1.5 mL of headspace vials into the detector of the electronic nose via a manual injection. A sample was measured 10 times, and data from 5 of these stable measurements were recorded for multivariate analysis (18).

### 2.3 E-tongue analysis

All samples were analyzed by an α-Astree electronic tongue (Alpha MOS, France) equipped with seven potentiometric sensors and an automatic sampler (19). The sensors were specifically sensitive to sweetness (ANS), saltiness (CTS), umami (NMS), sourness (AHS), bitterness (SCS) and two reference electrodes (PKS and CPS), respectively (20).

The crushed samples were diluted at a ratio of 1:10, followed by the addition of pure water for ultrasonic extraction lasting 10 min. Subsequently, the mixture was filtered. 80 mL of the sample was transferred into a 150 mL specific electronic tongue beaker for analysis. The signal acquisition time, stirring rate, and analysis duration were established at 120 s, 60 revolutions per min, and 3 min, correspondingly. Additionally, the metal probe attached to the sensor underwent thorough cleaning with deionized water for 1 min following each analysis prior to measurement. Response values were gathered within the time frame of 100–120 s based on the recommendations provided by Zhang et al. (11). The mean value of each sample served as the foundational data for robust principal component analysis (rPCA) in multivariate research.

### 2.4 HS-GC-IMS analysis

The HS-GC-IMS analysis was performed following the method of Wu et al. (17). The VOCs of SBD samples were characterized through a HS-GC-IMS system (Flavorspec®, G.A.S. Instrument, Munich, Germany) with a MXT-WAX capillary column (30 m × 0.53 mm × 1 μm) (Restek, Mount Ayr, United States). The crushed SBD sample, weighing precisely 2 g, was transferred to a 20 mL headspace vial equipped with a magnetic screw seal cover. Subsequently, the vial was incubated at 60°C for a duration of 10 min. An automated injection system injected exactly 1.5 mL of the headspace sample into the injector (without employing split mode), utilizing a heated syringe maintained at a temperature of 85°C. Drift gas flow rate was set at 150 mL/min. A high-purity N2 (99.99% purity) was utilized, and the GC column flow rate was programmed as follows: 2 mL/min for 5 min, 10 mL/min for 10 min, 15 mL/min for 5 min, 50 mL/min for 10 min, and 100 mL/min for 10 min. As reported in previous studies, it was determined that the retention index (RI) of volatile compounds was calculated using n-ketone C4–C9 as an external reference, compounds were identified by comparing the RI and ion drift time of the volatile compounds to the retention indices and drift times of the standards in the HS-GC-IMS library (8, 11). Additionally, GC-IMS allowed for the accurate detection and identification of some volatile monomers and dimers, which was impossible with GC–MS (21).

### 2.5 Free amino acid analysis (FAAs)

The determination of free amino acid levels in the SBD sample was carried out according to the method with slight adjustments as outlined by Xu et al. (22). The SBD sample weighing 3 g was accurately mixed with a measure of 20 mL sulfosalicylic acid (7 g/100 mL). The mixture was subjected to extraction for 30 min under ultrasonic vibration (KQ-300VDV, Kunshan Instrument, China) and subsequently centrifuged at 10,000×*g* for 15 min using the centrifuge (CR21N, Hitachi Koki Co., Ltd., Ibaraki, Japan). The supernatant was collected and later using a 0.22 μm filtered membrane. The determination of free amino acids was performed utilizing an Amino Acid Automatic (S-433D, SYKAM, Germany) Analyzer, Separation by sulfonic acid based strongly acidic cation exchange resin column (LCA K07/Li 150 mm × 4.6 mm), with identification and quantification achieved by comparing the retention times and peak areas of individual amino acid standards (Sigma-Aldrich, St. Louis, MO, USA) (23).

### 2.6 Relative odor activity value (ROAV)

The food contains numerous volatile organic compounds, but only a few significantly contribute to its flavor. These specific compounds, known as significant flavor compounds, are considered crucial for determining the overall flavor of the food (ROAV) (24). Based on the peak intensity characterization of HS-GC-IMS, the threshold value for each aroma compound in the sample was determined by referring to Xu et al.’s research methodology (25). Then, the relative odor activity value (ROAV) of each aroma compound was calculated as follows:


$$\mathrm{ROAV}=100\times \frac{C_{i}}{C_{\text{max}}}\times \frac{T_{\text{max}}}{T_{i}}$$

In the formula, *C_i_* is the relative content of the aroma compound in samples (%); *T_i_* is the aroma threshold of the compound in food (μg/kg), *C*_max_ and *T*_max_ represent the relative content and aroma threshold of the compound that contributes the most to the overall flavor of the sample. For all compounds, ROAV ≤ 100, A higher ROAV value indicates a more significant contribution of the component to the overall flavor profile of the model (26, 27).

### 2.7 Taste activity value (TAV)

The TAV has emerged as a widely accepted and effective method for evaluating taste in recent years (22). It involves calculating the ratio between the concentration of a taste substance and its taste threshold, allowing us to determine the contribution of compounds with free amino acid taste characteristics to the overall taste perception (28). TAV is calculated as follows:


$$TAV=\frac{w_{1}}{w_{2}}$$

In the formula, *w*_1_ is the compound content in the sample/(mg/100 g), and *w*_2_ is the compound/(mg/100 g) taste threshold. A TAV ≥ 1 for an amino acid indicates that it is a significant contributor to the overall taste experience, while an amino acid with 0.1 ≤ TAV ≤ 1 is considered to have an essential modifying effect on taste (13).

### 2.8 Statistical analysis

The statistical analysis was performed using the R language. Before conducting univariate analyses, the data distribution was transformed by the Box and Cox method to achieve normality (29). ANOVA was performed to find significant differences in the E-nose and E-tongue sensor’s response to aroma and taste from different groups, respectively, followed by the Tukey HSD post-hoc test (*p* < 0.05) (30). For each rPCA model, we computed a score plot and a Pearson correlation plot based on the loadings (31). The three-dimensional (3D) topographic plots, two-dimensional (2D) difference plots, and gallery plots were generated using the Laboratory Analytical Viewer, Reporter and Gallery Plot provided by the HS-GC-IMS instrument. Using online tools.^1^ Through making a heat map to determine the vital components of the SBD.

## 3 Results

### 3.1 Intelligent senses analysis of Steamed beef with rice flour

#### 3.1.1 E-nose analysis of SBD

**Figure 2:**
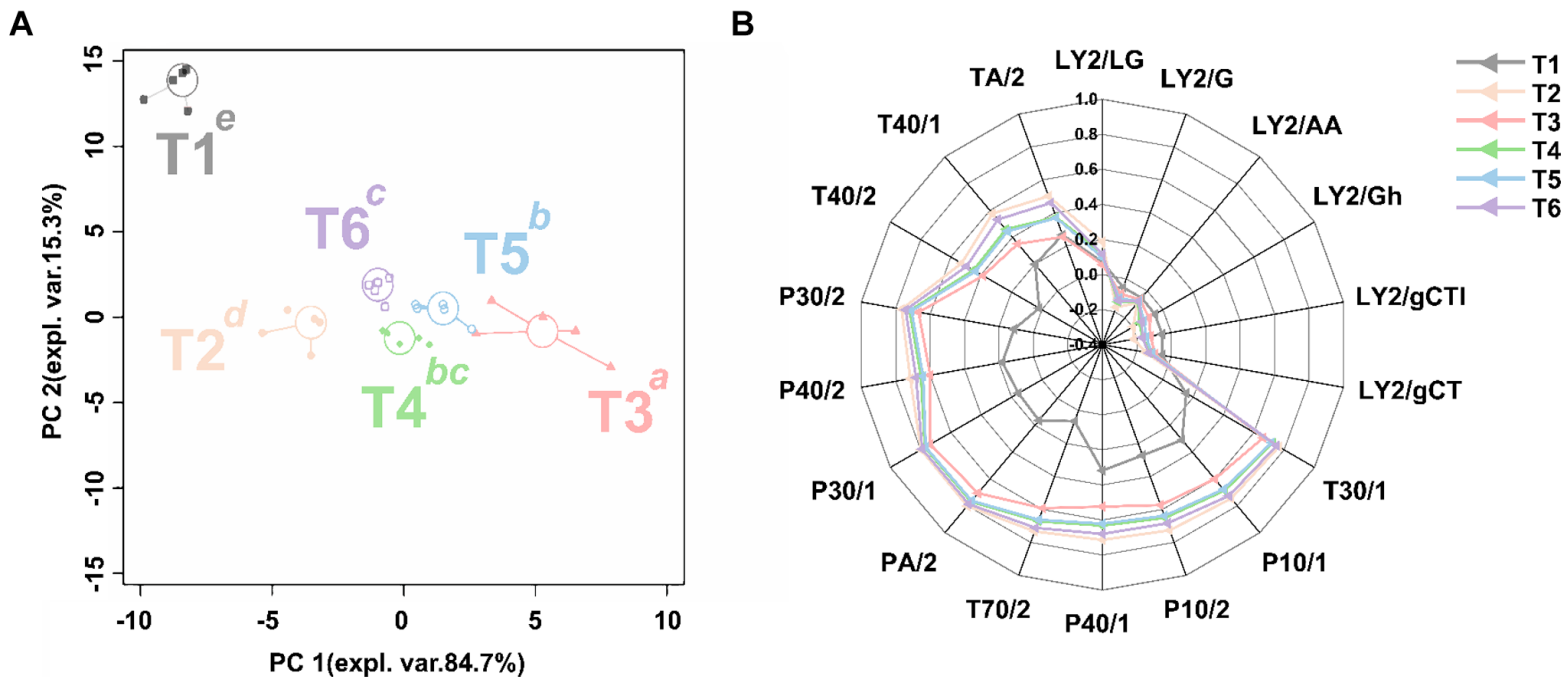
The rPCA model based on the response value of E-nose sensor. The score plot **(A)** illustrates the overall trend of the samples. Lowercase superscript letters indicate significant differences (*p* < 0.05) between samples on PC1. The radar chart **(B)** illustrates the response value of the sensor on PC1.

In this study, the rPCA model is established to describe the aroma characteristics of the SBD. The proportion of PC 1 in the entire model, as depicted in Figure 2A, amounts to 84.7%, exhibiting significant variations among samples at different stages (*p* < 0.05). Based on the intensity of the 18 sensors’ responses to a specific characteristic gas, the main characteristic gas in each stage was tentatively speculated. As shown in Figure 2B, the response of each sensor was lower in the raw meat sample (T1), while the organic compounds (PA/2, P30/1, P30/2) and gases with high oxidizing power (P40/1, P40/2, T40/2, T40/1), polar compounds (T30/1); non-polar compounds (P10/1), alkanes (P10/2), aromatic compounds (T70/2) increased during the seasoning stage (T3). In contrast, gases with high oxidizing power (T40/1, T40/2, LY2/LG) and while the organic compounds were the main contributors in the steaming stage (T2), followed by organic compounds (TA/2). Furthermore, as the steaming process progresses, the sensor response values of the electronic nose gradually increase, reaching their highest point at the end of the steaming process (T6). Steaming for 20 min (T4) and 40 min (T5) resulted in the predominant gases being organic compounds, high oxidizing power gases, aromatic compounds, and alkanes (TA/2, T40/1, T40/2, P40/1, P40/2, T70/2, P10/1, P10/2), with these compounds peaking at the end of the steaming process (T6). These results indicate that the odor of SBD underwent significant changes during the marinating, seasoning stage, and steaming process.

#### 3.1.2 E-tongue analysis of SBD

**Figure 3:**
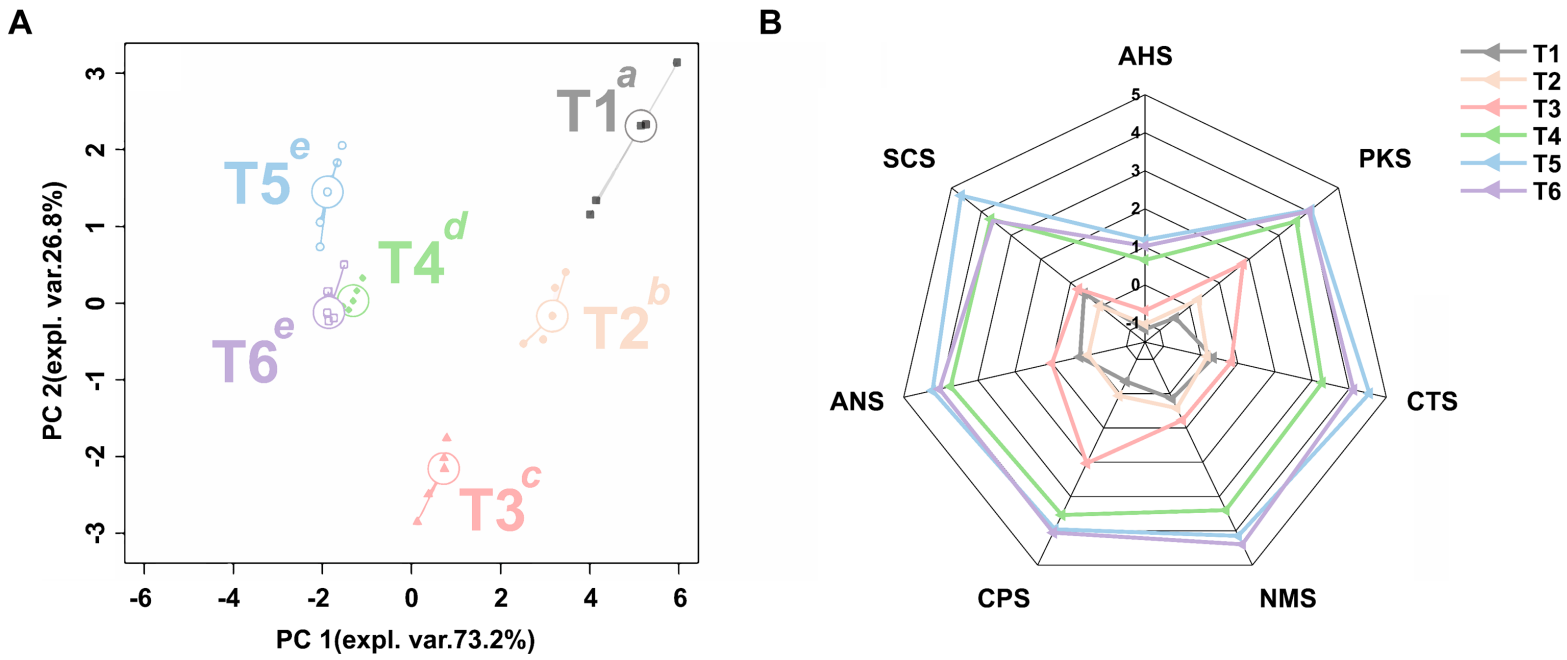
The rPCA model based on the response value of electronic tongue sensor. The score plot **(A)** illustrates the overall trend of the samples. Lowercase superscript letters indicate significant differences (*p* < 0.05) between samples on PC1. The radar chart **(B)** illustrates the response value of the sensor on PC1.

To gain a comprehensive understanding of the taste characteristics of SBD during the production process, an rPCA model was established based on the response values obtained from the seven sensors of the electronic tongue, as illustrated in Figure 3. PC1 accounts for 73.2% of the sample, better summarizing the sample differences. Based on the intensity of the seven sensors’ responses to a specific characteristic, the main taste characteristics in each stage was tentatively speculated. As shown in Figure 3A, the electronic tongue sensor response values of samples at different stages in the SBD have significant differences on PC1 (*p* < 0.05). As shown in Figure 3B, in the raw meat sample (T1), the response value of each sensor was low, but with the progress of cooking, the response value of the electronic tongue sensor increases. After removing the reference electrode (CPS, PKS), the bitter, sweet, umami, and salty sensors (SCS, ANS, CTS) become the main feature during the marinating stage (T2). And the electronic tongue sensor responses further increase during the seasoning stage (T3). It is worth noting that at 20 min (T4), into the steaming process, all the taste components began to gradually increase, and at 40 min (T5) into the steaming process, the bitterness (SCS) and saltiness (CTS) of SBD were the most intense. Then, at the end of cooking (T6), the umami (NMS) is the most intense in the SBD.

### 3.2 Volatile compounds were analyzed by HS-GC-IMS

#### 3.2.1 HS-GC-IMS topographic map of SBD

**Figure 4:**
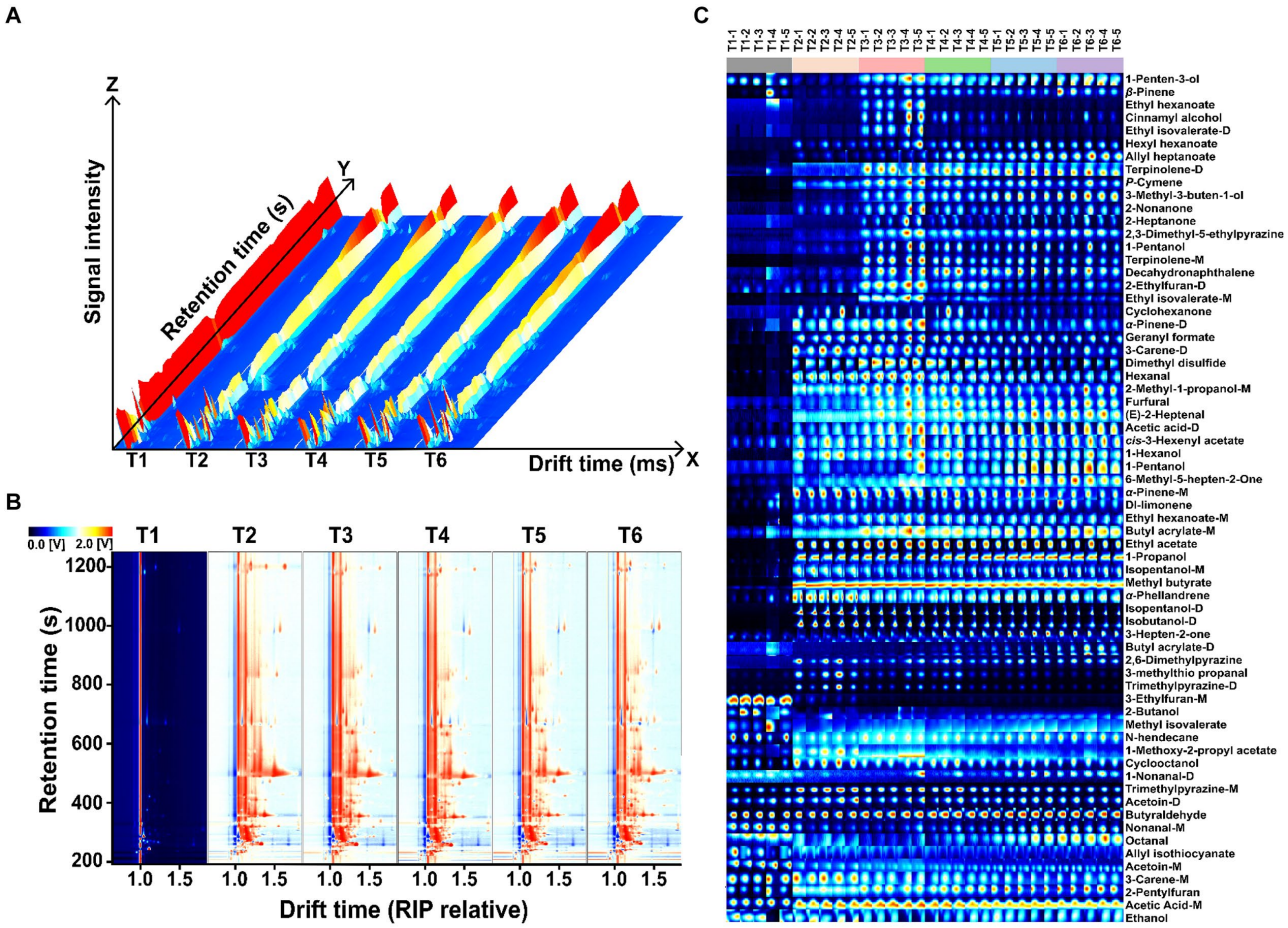
Presents the GC-IMS diagram of SBD at various processing stages. **(A)** 3D topographic maps, **(B)** gallery plot indicating changes in VOCs concentration across different stages, and **(C)** 2D difference map illustrating the variation from the control sample T1 by subtracting spectra of T2-T6. The color scheme employs red and blue to represent high or low compound concentrations, respectively.

The processing’s pipeline of HS-GC-IMS information on the VOCs in the SBD by during the stages is summarized in Figure 4. The 3D topographic map conveniently and intuitively shows the difference of GC-IMS spectrum of SBD in the production process (Figure 4A). In the SBD, the levels of VOCs in stages T2–T6 were significantly elevated compared to the control group T1. To visually highlight the variations in VOCs among the samples, choosing the difference map illustrates 2D representation (Figure 4B) for comparison. Specifically, compared with T1, the red area in the SBD after processing has increased, among which T4–T6 have a similar red area. The peak intensity of VOCs was the highest when SBD was produced in the T3 stage. In the gallery plot (Figure 4C), it can be seen that VOCs in SBD samples at different processing stages mainly include esters and alcohols. VOCs of the SBD were qualitatively analyzed according to gas chromatography retention time and ion migration time, and the results were shown in Table 1, 66 volatile organic compounds (mono-polymers and di-polymers) were characterized in the six samples, including esters (15), alcohols (14), olefins (9), aldehydes (8), ketones (7), heterocyclic (7), acids (2), and others (4).

**Table 1:** The peak intensity (mean ± sd) of volatile organic compounds in different SBD stages was characterized by GC-IMS.

Count	Compounds	CAS	Formula	RI^#^	DT[ms]	Peak intensity					
						T1	T2	T3	T4	T5	T6
Esters	Ethyl Acetate	141-78-6	C_4_H_8_O_2_	904.9	1.32917	2.53 × 10^2^ ± 1.30 × 10^2 c *^	5.22 × 10^3^ ± 7.24 × 10^2 b^	7.22 × 10^3^ ± 1.38 × 10^2 a^	6.25 × 10^3^ ± 5.57 × 10^2 ab^	5.38 × 10^3^ ± 3.67 × 10^2 b^	5.13 × 10^3^ ± 9.29 × 10^2 b^
	Ethyl hexanoate-M	123-66-0	C_8_H_16_O_2_	1,213.6	1.32158	9.62 × 10^2^ ± 8.53 × 10^2 c^	9.04 × 10^3^ ± 5.87 × 10^2 a^	9.48 × 10^3^ ± 4.17 × 10^2 a^	8.04 × 10^3^ ± 5.41 × 10^2 ab^	7.84 × 10^3^ ± 3.39 × 10^2 ab^	7.30 × 10^3^ ± 6.61 × 10^2 b^
	Ethyl 3-methylbutanoate-M	108-64-5	C_7_H_14_O_2_	1,064.6	1.26613	1.23 × 10^2^ ± 4.73 × 10^1 d^	2.39 × 10^2^ ± 2.49 × 10^1 c^	1.32 × 10^3^ ± 8.13 × 10^1 a^	6.39 × 10^2^ ± 1.92 × 10^2 b^	4.44 × 10^2^ ± 3.76 × 10^1 b^	4.78 × 10^2^ ± 6.84 × 10^1 b^
	Ethyl 3-methylbutanoate-D	108-64-5	C_7_H_14_O_2_	1,065.0	1.65372	1.72 × 10^2^ ± 4.68 × 10^1 b^	1.13 × 10^2^ ± 2.10 × 10^1 c^	5.54 × 10^2^ ± 9.83 × 10^1 a^	1.70 × 10^2^ ± 4.54 × 10^1 b^	1.11 × 10^2^ ± 6.52 × 10^0 bc^	1.31 × 10^2^ ± 1.45 × 10^1 bc^
	Butyl 2-propenoate-M	141-32-2	C_7_H_12_O_2_	1,186.9	1.26163	1.25 × 10^2^ ± 2.91 × 10^1 d^	2.07 × 10^2^ ± 3.08 × 10^1 c^	2.73 × 10^2^ ± 2.71 × 10^1 bc^	2.57 × 10^2^ ± 4.93 × 10^1 bc^	3.08 × 10^2^ ± 2.10 × 10^1 ab^	3.54 × 10^2^ ± 3.29 × 10^1 a^
	Butyl 2-propenoate-D	141-32-2	C_7_H_12_O_2_	1,186.0	1.6939	1.42 × 10^2^ ± 7.30 × 10^1 bc^	8.96 × 10^1^ ± 9.39 × 10^0 d^	1.12 × 10^2^ ± 2.07 × 10^1 cd^	1.36 × 10^2^ ± 3.10 × 10^1 bc^	1.83 × 10^2^ ± 1.00 × 10^1 ab^	3.06 × 10^2^ ± 7.88 × 10^1 a^
	Geranyl formate	105-86-2	C_11_H_18_O_2_	1,292.9	1.21058	1.02 × 10^3^ ± 1.09 × 10^2 d^	4.83 × 10^3^ ± 5.40 × 10^2 ab^	5.01 × 10^3^ ± 4.78 × 10^2 a^	4.16 × 10^3^ ± 5.06 × 10^2 bc^	4.04 × 10^3^ ± 1.81 × 10^2 bc^	3.77 × 10^3^ ± 2.85 × 10^2 c^
	(Z)-3-Hexenyl acetate	3681-71-8	C_8_H_14_O_2_	1,362.8	1.32946	1.47 × 10^2^ ± 1.83 × 10^1 b^	2.83 × 10^2^ ± 4.18 × 10^1 a^	3.28 × 10^2^ ± 1.70 × 10^1 a^	2.64 × 10^2^ ± 3.88 × 10^1 a^	2.92 × 10^2^ ± 2.42 × 10^1 a^	3.12 × 10^2^ ± 3.94 × 10^1 a^
	Methyl butyrate	623-42-7	C_5_H_10_O_2_	990.5	1.12459	6.99 × 10^2^ ± 8.54 × 10^1 c^	7.32 × 10^3^ ± 1.43 × 10^2 a^	7.20 × 10^3^ ± 7.01 × 10^1 a^	6.99 × 10^3^ ± 2.26 × 10^2 ab^	6.82 × 10^3^ ± 2.49 × 10^2 b^	6.63 × 10^3^ ± 2.43 × 10^2 b^
	Isovaleric acid, methyl	556-24-1	C_6_H_12_O_2_	1,017.1	1.19216	2.54 × 10^2^ ± 1.40 × 10^2 a^	8.42 × 10^1^ ± 1.97 × 10^1 c^	1.13 × 10^2^ ± 3.92 ^bc^	1.07 × 10^2^ ± 1.09 × 10^1 bc^	1.58 × 10^2^ ± 2.13 × 10^1 ab^	1.39 × 10^2^ ± 1.07 × 10^1 b^
	1-Methoxy-2-propanol acetate	108-65-6	C_6_H_12_O_3_	1,234.1	1.17298	1.35 × 10^2^ ± 2.67 × 10^1 c^	2.23 × 10^2^ ± 4.11 × 10^1 a^	2.14 × 10^2^ ± 4.38 × 10^1 ab^	1.46 × 10^2^ ± 2.57 × 10^1 bc^	1.08 × 10^2^ ± 2.05 × 10^1 cd^	9.66 × 10^1^ ± 2.42 × 10^1 d^
	Allyl heptanoate	142-19-8	C_10_H_18_O_2_	1,188.5	1.4365	1.06 × 10^2^ ± 5.24 × 10^1 d^	3.90 × 10^2^ ± 8.92 × 10^1 c^	5.17 × 10^2^ ± 1.39 × 10^2 c^	9.02 × 10^2^ ± 1.89 × 10^2 b^	1.26 × 10^3^ ± 1.61 × 10^2 a^	1.42 × 10^3^ ± 2.00 × 10^2 a^
	Hexyl hexanoate	6378-65-0	C_12_H_24_O_2_	1,395.4	1.57128	3.60 × 10^2^ ± 5.69 × 10^1 b^	1.24 × 10^3^ ± 4.13 × 10^2 a^	2.00 × 10^3^ ± 1.04 × 10^3 a^	1.64 × 10^3^ ± 4.21 × 10^2 a^	1.81 × 10^3^ ± 3.34 × 10^2 a^	1.81 × 10^3^ ± 2.39 × 10^2 a^
	Ethyl hexanoate-D	123-66-0	C_8_H_16_O_2_	1,227.1	1.78994	1.48 × 10^2^ ± 1.47 × 10^2 b^	9.75 × 10^1^ ± 2.65 × 10^1 b^	3.70 × 10^2^ ± 1.07 × 10^2 a^	7.13 × 10^1^ ± 1.23 × 10^1 b^	7.66 × 10^1^ ± 6.84 ^b^	8.38 × 10^1^ ± 9.49 ^b^
	Allyl Isothiocyanate	57-06-7	C_4_H_5_NS	930.0	1.0915	1.06 × 10^3^ ± 3.73 × 10^2 a^	8.68 × 10^2^ ± 7.42 × 10^1 ab^	7.50 × 10^2^ ± 3.26 × 10^1 ab^	6.25 × 10^2^ ± 5.36 × 10^1 bc^	6.14 × 10^2^ ± 2.71 × 10^1 bc^	5.22 × 10^2^ ± 2.01 × 10^1 c^
Alcohols	2-Methyl-1-propanol-D	78-83-1	C_4_H_10_O	1,095.7	1.37398	3.63 × 10^2^ ± 1.05 × 10^2 d^	6.89 × 10^3^ ± 5.28 × 10^2 a^	5.20 × 10^3^ ± 9.09 × 10^1 b^	4.80 × 10^3^ ± 3.77 × 10^2 bc^	4.47 × 10^3^ ± 2.37 × 10^2 c^	4.09 × 10^3^ ± 5.22 × 10^2 c^
	3-Methylbutanol-D	123-51-3	C_5_H_12_O	1,204.7	1.51194	2.95 × 10^3^ ± 3.65 × 10^2 e^	2.16 × 10^4^ ± 1.93 × 10^3 a^	1.50 × 10^4^ ± 7.57 × 10^2 b^	1.20 × 10^4^ ± 1.37 × 10^3 c^	1.06 × 10^4^ ± 9.82 × 10^2 cd^	9.66 × 10^3^ ± 1.02 × 10^3 d^
	3-Methylbutanol-M	123-51-3	C_5_H_12_O	1,203.5	1.25161	7.11 × 10^2^ ± 3.15 × 10^2 c^	6.35 × 10^3^ ± 3.73 × 10^2 a^	4.30 × 10^3^ ± 4.94 × 10^2 b^	4.46 × 10^3^ ± 1.11 × 10^2 b^	4.51 × 10^3^ ± 9.26 × 10^1 b^	4.57 × 10^3^ ± 1.64 × 10^2 b^
	Pentanol-M	71-41-0	C_5_H_12_O	1,296.1	1.50898	1.05 × 10^2^ ± 1.24 × 10^1 d^	1.81 × 10^2^ ± 9.09 × 10^1 cd^	2.24 × 10^2^ ± 1.15 × 10^2 c^	2.68 × 10^2^ ± 5.25 × 10^1 bc^	4.00 × 10^2^ ± 6.13 × 10^1 ab^	4.76 × 10^2^ ± 8.23 × 10^1 a^
	Pentanol-D	71-41-0	C_5_H_12_O	1,250.3	1.51605	1.05 × 10^2^ ± 1.77 × 10^1 c^	1.89 × 10^2^ ± 4.09 × 10^1 b^	4.21 × 10^2^ ± 6.01 × 10^1 a^	3.03 × 10^2^ ± 7.89 × 10^1 a^	3.49 × 10^2^ ± 4.56 × 10^1 a^	3.39 × 10^2^ ± 8.80 × 10^1 a^
	3-Methyl-3-buten-1-ol	763-32-6	C_5_H_10_O	1,311.2	1.16675	1.36 × 10^2^ ± 1.75 × 10^1 d^	4.06 × 10^2^ ± 7.18 × 10^1 c^	1.70 × 10^3^ ± 1.91 × 10^2 ab^	1.48 × 10^3^ ± 1.45 × 10^2 b^	1.50 × 10^3^ ± 6.37 × 10^1 b^	1.88 × 10^3^ ± 2.63 × 10^2 a^
	1-Penten-3-ol	616-25-1	C_5_H_10_O	1,181.2	1.35689	4.27 × 10^2^ ± 9.54 × 10^1 b^	2.16 × 10^2^ ± 4.83 × 10^1 c^	7.19 × 10^2^ ± 1.21 × 10^2 a^	6.39 × 10^2^ ± 1.25 × 10^2 a^	6.19 × 10^2^ ± 2.51 × 10^1 a^	7.69 × 10^2^ ± 1.33 × 10^2 a^
	1-Hexanol	111-27-3	C_6_H_14_O	1,349.2	1.30244	1.46 × 10^2^ ± 2.71 × 10^1 b^	7.84 × 10^2^ ± 2.37 × 10^2 a^	7.86 × 10^2^ ± 9.88 × 10^1 a^	7.97 × 10^2^ ± 1.63 × 10^2 a^	7.88 × 10^2^ ± 6.73 × 10^1 a^	7.86 × 10^2^ ± 1.02 × 10^2 a^
	2-butanol	78-92-2	C_4_H_10_O	1,024.6	1.29733	1.45 × 10^2^ ± 6.23 × 10^1 a^	8.51 × 10^1^ ± 3.57 × 10^1 ab^	7.96 × 10^1^ ± 2.93 × 10^1 b^	6.94 × 10^1^ ± 5.96 × 10^0 b^	9.57 × 10^1^ ± 1.44 × 10^1 ab^	1.00 × 10^2^ ± 1.15 × 10^1 ab^
	Propanol	71-23-8	C_3_H_8_O	1,031.8	1.11502	6.01 × 10^2^ ± 6.09 × 10^1 c^	7.95 × 10^3^ ± 8.75 × 10^2 b^	7.96 × 10^3^ ± 8.52 × 10^2 b^	8.38 × 10^3^ ± 9.31 × 10^2 b^	1.09 × 10^4^ ± 5.55 × 10^2 a^	9.40 × 10^3^ ± 8.97 × 10^2 ab^
	Ethanol	64-17-5	C_2_H_6_O	913.9	1.06125	3.32 × 10^2^ ± 1.62 × 10^2 a^	3.35 × 10^2^ ± 5.26 × 10^1 a^	2.77 × 10^2^ ± 7.46 × 10^1 a^	2.89 × 10^2^ ± 6.50 × 10^1 a^	3.31 × 10^2^ ± 5.61 × 10^1 a^	3.47 × 10^2^ ± 4.90 × 10^1 a^
	2-Methyl-1-propanol-M	78-83-1	C_4_H_10_O	1,107	1.17449	4.31 × 10^1^ ± 7.06 × 10^0 e^	5.52 × 10^2^ ± 4.14 × 10^1 bc^	7.00 × 10^2^ ± 2.19 × 10^1 a^	5.67 × 10^2^ ± 3.54 × 10^1 b^	4.05 × 10^2^ ± 3.80 × 10^1 d^	4.65 × 10^2^ ± 8.84 × 10^1 cd^
	Cinnamyl alcohol	104-54-1	C_9_H_10_O	1,274.5	1.56659	5.10 × 10^1^ ± 8.00 ^c^	5.04 × 10^1^ ± 4.91 ^c^	2.82 × 10^2^ ± 6.41 × 10^1 a^	1.42 × 10^2^ ± 3.99 × 10^1 b^	9.76 × 10^1^ ± 1.70 × 10^1 b^	1.09 × 10^2^ ± 4.47 × 10^1 b^
	Cyclooctanol	696-71-9	C_8_H_16_O	1,148.8	1.12058	1.23 × 10^3^ ± 3.68 × 10^2 b^	3.69 × 10^3^ ± 4.46 × 10^2 a^	3.29 × 10^3^ ± 2.62 × 10^2 a^	3.44 × 10^3^ ± 2.44 × 10^2 a^	3.04 × 10^3^ ± 2.95 × 10^2 a^	2.94 × 10^3^ ± 2.51 × 10^2 a^
Alkenes	Limonene	138-86-3	C_10_H_16_	1,206.8	1.21404	4.14 × 10^2^ ± 4.51 × 10^2 b^	6.71 × 10^2^ ± 2.20 × 10^2 ab^	6.21 × 10^2^ ± 2.22 × 10^1 ab^	9.94 × 10^2^ ± 2.71 × 10^2 a^	1.03 × 10^3^ ± 7.90 × 10^1 a^	1.14 × 10^3^ ± 4.53 × 10^2 a^
	α-pinene-M	80-56-8	C_10_H_16_	1,033.7	1.20957	2.29 × 10^2^ ± 1.17 × 10^2 d^	1.49 × 10^3^ ± 1.67 × 10^2 ab^	1.60 × 10^3^ ± 4.15 × 10^1 a^	1.42 × 10^3^ ± 1.70 × 10^2 ab^	1.06 × 10^3^ ± 1.10 × 10^2 c^	1.17 × 10^3^ ± 1.57 × 10^2 bc^
	α-pinene-D	80-56-8	C_10_H_16_	1,033.7	1.31662	2.23 × 10^1^ ± 4.44 ^d^	1.50 × 10^2^ ± 2.68 × 10^1 ab^	1.82 × 10^2^ ± 1.33 × 10^1 a^	1.22 × 10^2^ ± 3.01 × 10^1 b^	7.93 × 10^1^ ± 7.03 ^c^	8.23 × 10^1^ ± 2.49 × 10^1 c^
	3-Carene-D	13466-78-9	C_10_H_16_	1,139.2	1.26709	6.20 × 10^1^ ± 9.71 × 10^1 d^	9.33 × 10^2^ ± 1.17 × 10^2 a^	9.20 × 10^2^ ± 9.53 × 10^1 a^	6.91 × 10^2^ ± 1.13 × 10^2 b^	5.31 × 10^2^ ± 5.33 × 10^1 bc^	4.81 × 10^2^ ± 8.49 × 10^1 c^
	3-Carene-M	13466-78-9	C_10_H_16_	1,140.1	1.1892	3.55 × 10^2^ ± 4.13 × 10^1 ab^	4.44 × 10^2^ ± 3.50 × 10^1 a^	3.51 × 10^2^ ± 7.95 × 10^0 ab^	3.11 × 10^2^ ± 4.03 × 10^1 bc^	2.53 × 10^2^ ± 1.86 × 10^1 d^	2.64 × 10^2^ ± 3.59 × 10^1 cd^
	Terpinolene-D	586-62-9	C_10_H_16_	1,279.4	1.28723	1.37 × 10^2^ ± 4.14 × 10^1 b^	1.21 × 10^2^ ± 5.04 ^b^	3.59 × 10^2^ ± 3.79 × 10^1 a^	3.34 × 10^2^ ± 5.64 × 10^1 a^	4.11 × 10^2^ ± 2.46 × 10^1 a^	4.07 × 10^2^ ± 5.42 × 10^1 a^
	Terpinolene-M	586-62-9	C_10_H_16_	1,279.9	1.21944	8.83 × 10^1^ ± 1.31 × 10^1 d^	1.64 × 10^2^ ± 1.56 × 10^1 c^	1.36 × 10^3^ ± 7.73 × 10^1 a^	9.67 × 10^2^ ± 1.97 × 10^2 b^	9.25 × 10^2^ ± 1.16 × 10^2 b^	8.41 × 10^2^ ± 2.01 × 10^2 b^
	β-pinene	127-91-3	C_10_H_16_	1,116.6	1.21818	4.82 × 10^2^ ± 6.17 × 10^2 bc^	2.73 × 10^2^ ± 5.88 × 10^1 c^	9.95 × 10^2^ ± 9.42 × 10^1 a^	7.55 × 10^2^ ± 1.42 × 10^2 ab^	8.31 × 10^2^ ± 6.00 × 10^1 ab^	1.14 × 10^3^ ± 3.04 × 10^2 a^
	α-phellandrene	99-83-2	C_10_H_16_	1,161	1.21523	5.28 × 10^2^ ± 5.41 × 10^2 a^	3.65 × 10^2^ ± 9.28 × 10^1 a^	3.27 × 10^2^ ± 3.50 × 10^1 a^	3.44 × 10^2^ ± 1.36 × 10^2 a^	3.44 × 10^2^ ± 2.87 × 10^1 a^	3.61 × 102 ± 1.09 × 10^2 a^
Aldehydes	Nonanal-M	124-19-6	C_9_H_18_O	1,395.2	1.49576	1.85 × 10^3^ ± 7.25 × 10^1 a^	9.19 × 10^2^ ± 2.99 × 10^2 c^	9.78 × 10^2^ ± 3.80 × 10^2 bc^	1.04 × 10^3^ ± 2.63 × 10^2 bc^	1.44 × 10^3^ ± 1.99 × 10^2 ab^	1.62 × 10^3^ ± 7.58 × 10^1 a^
	3-Methylthio propanal	3268-49-3	C_4_H_8_OS	1,449.1	1.39672	2.37 × 10^2^ ± 3.61 × 10^1 c^	1.20 × 10^3^ ± 4.93 × 10^2 a^	6.04 × 10^2^ ± 1.63 × 10^2 b^	6.21 × 10^2^ ± 2.56 × 10^2 b^	3.86 × 10^2^ ± 5.14 × 10^1 bc^	3.03 × 10^2^ ± 4.01 × 10^1 c^
	Octanal	124-13-0	C_8_H_16_O	1,295.5	1.41812	2.76 × 10^2^ ± 4.10 × 10^1 a^	1.75 × 10^2^ ± 3.56 × 10^1 b^	1.29 × 10^2^ ± 2.57 × 10^1 b^	1.68 × 10^2^ ± 2.70 × 10^1 b^	3.26 × 10^2^ ± 2.84 × 10^1 a^	3.67 × 10^2^ ± 4.24 × 10^1 a^
	(E)-2-Heptenal	18829-55-5	C_7_H_12_O	1,333.4	1.25527	8.51 × 10^1^ ± 1.48 × 10^1 d^	2.69 × 10^2^ ± 1.26 × 10^1 c^	2.93 × 10^2^ ± 1.15 × 10^1 bc^	2.99 × 10^2^ ± 3.08 × 10^1 bc^	3.44 × 10^2^ ± 3.63 × 10^1 ab^	3.58 × 10^2^ ± 3.00 × 10^1 a^
	Furfural	98-01-1	C_5_H_4_O_2_	1,445.7	1.08713	3.50 × 10^2^ ± 1.35 × 10^1 b^	3.54 × 10^2^ ± 2.66 × 10^1 b^	6.14 × 10^2^ ± 8.08 × 10^1 a^	6.31 × 10^2^ ± 1.32 × 10^2 a^	6.08 × 10^2^ ± 4.09 × 10^1 a^	6.85 × 10^2^ ± 6.16 × 10^1 a^
	Nonanal-D	124-19-6	C_9_H_18_O	1,395.9	1.93198	2.68 × 10^2^ ± 3.96 × 10^1 ab^	1.92 × 10^2^ ± 2.90 × 10^1 b^	2.49 × 10^2^ ± 1.28 × 10^2 ab^	2.31 × 10^2^ ± 6.32 × 10^1 ab^	2.92 × 10^2^ ± 5.28 × 10^1 a^	3.39 × 10^2^ ± 6.24 × 10^1 a^
	Hexanal	66-25-1	C_6_H_12_O	1,088.8	1.25499	3.15 × 10^2^ ± 4.01 × 10^1 e^	3.45 × 10^3^ ± 1.42 × 10^2 a^	3.41 × 10^3^ ± 8.85 × 10^1 a^	2.84 × 10^3^ ± 1.50 × 10^2 b^	2.56 × 10^3^ ± 8.60 × 10^1 c^	2.30 × 10^3^ ± 1.61 × 10^2 d^
	Butyraldehyde	123-72-8	C_4_H_8_O	854.3	1.11424	8.37 × 10^3^ ± 8.94 × 10^2 c^	9.94 × 10^3^ ± 6.75 × 10^2 b^	1.22 × 10^4^ ± 4.24 × 10^2 a^	1.33 × 10^4^ ± 6.53 × 10^2 a^	1.31 × 10^4^ ± 4.37 × 10^2 a^	1.28 × 10^4^ ± 3.99 × 10^2 a^
Ketones	3-Hydroxy-2-butanone-D	513-86-0	C_4_H_8_O_2_	1,293.6	1.33027	1.56 × 10^3^ ± 2.64 × 10^2 b^	2.35 × 10^3^ ± 3.55 × 10^2 a^	1.69 × 10^3^ ± 1.61 × 10^2 ab^	1.62 × 10^3^ ± 3.83 × 10^2 b^	1.92 × 10^3^ ± 1.32 × 10^2 ab^	1.82 × 10^3^ ± 9.65 × 10^1 ab^
	3-Hydroxy-2-butanone-M	513-86-0	C_4_H_8_O_2_	1,297.9	1.08651	5.55 × 10^3^ ± 4.70 × 10^2 a^	2.60 × 10^3^ ± 1.19 × 10^2 bc^	2.27 × 10^3^ ± 1.58 × 10^2 cd^	2.22 × 10^3^ ± 2.11 × 10^2 d^	2.66 × 10^3^ ± 1.00 × 10^2 b^	2.84 × 10^3^ ± 1.79 × 10^2 b^
	6-Methyl-5-hepten-2-one	110-93-0	C_8_H_14_O	1,343.5	1.17549	8.54 × 10^1^ ± 1.12 × 10^1 c^	2.06 × 10^2^ ± 4.35 × 10^1 b^	3.15 × 10^2^ ± 7.95 × 10^1 a^	3.45 × 10^2^ ± 5.88 × 10^1 a^	3.56 × 10^2^ ± 5.20 × 10^1 a^	4.19 × 10^2^ ± 4.32 × 10^1 a^
	Cyclohexanone	108-94-1	C_6_H_10_O	1,289.7	1.46084	8.52 × 10^1^ ± 1.45 × 10^1 b^	2.16 × 10^2^ ± 7.44 × 10^1 a^	1.35 × 10^2^ ± 2.37 × 10^1 ab^	1.83 × 10^2^ ± 7.18 × 10^1 a^	1.38 × 10^2^ ± 2.07 × 10^1 a^	1.41 × 10^2^ ± 2.06 × 10^1 a^
	2-Nonanone	821-55-6	C_9_H_18_O	1,363.7	1.4139	1.04 × 10^2^ ± 1.36 × 10^1 c^	2.02 × 10^2^ ± 3.80 × 10^1 b^	3.82 × 10^2^ ± 5.38 × 10^1 a^	2.65 × 10^2^ ± 4.54 × 10^1 b^	2.74 × 10^2^ ± 2.67 × 10^1 b^	2.81 × 10^2^ ± 6.00 × 10^1 b^
	2-Heptanone	110-43-0	C_7_H_14_O	1,176.2	1.63167	1.14 × 10^2^ ± 3.77 × 10^1 b^	1.00 × 10^2^ ± 7.55 × 10^0 b^	3.41 × 10^2^ ± 1.44 × 10^2 a^	2.30 × 10^2^ ± 4.61 × 10^1 a^	2.32 × 10^2^ ± 2.10 × 10^1 a^	2.78 × 10^2^ ± 7.05 × 10^1 a^
	3-Hepten-2-one	1119-44-4	C_7_H_12_O	917.4	1.22565	1.38 × 10^3^ ± 1.14 × 10^2 d^	2.36 × 10^3^ ± 1.78 × 10^2 c^	3.01 × 10^3^ ± 2.09 × 10^2 b^	3.59 × 10^3^ ± 3.12 × 10^2 a^	3.85 × 10^3^ ± 3.28 × 10^2 a^	3.51 × 10^3^ ± 1.73 × 10^2 a^
Heterocycles	2,3,5-Trimethylpyrazine-D	14667-55-1	C_7_H_10_N_2_	1,448.6	1.63356	6.36 × 10^2^ ± 8.14 × 10^1 c^	2.91 × 10^3^ ± 1.13 × 10^3 a^	1.52 × 10^3^ ± 3.74 × 10^2 b^	1.57 × 10^3^ ± 6.56 × 10^2 b^	9.96 × 10^2^ ± 1.35 × 10^2 bc^	7.95 × 10^2^ ± 6.08 × 10^1 c^
	2,3,5-Trimethylpyrazine-M	14667-55-1	C_7_H_10_N_2_	1,447.9	1.18318	2.32 × 10^3^ ± 3.99 × 10^2 c^	5.46 × 10^3^ ± 8.83 × 10^2 a^	4.02 × 10^3^ ± 3.50 × 10^2 ab^	4.24 × 10^3^ ± 8.87 × 10^2 ab^	3.58 × 10^3^ ± 3.78 × 10^2 b^	3.15 × 10^3^ ± 2.81 × 10^2 b^
	2,6-Dimethylpyrazine	108-50-9	C_6_H_8_N_2_	1,351.2	1.53163	2.55 × 10^2^ ± 1.46 × 10^1 b^	7.51 × 10^2^ ± 2.17 × 10^2 a^	5.93 × 10^2^ ± 9.90 × 10^1 a^	6.56 × 10^2^ ± 1.68 × 10^2 a^	6.61 × 10^2^ ± 5.82 × 10^1 a^	7.32 × 10^2^ ± 8.75 × 10^1 a^
	2-Pentylfuran	3777-69-3	C_9_H_14_O	1,243.3	1.25673	4.29 × 10^2^ ± 9.25 × 10^1 b^	6.22 × 10^2^ ± 2.29 × 10^1 a^	6.84 × 10^2^ ± 4.17 × 10^1 a^	6.78 × 10^2^ ± 4.17 × 10^1 a^	6.22 × 10^2^ ± 5.63 × 10^1 a^	6.95 × 10^2^ ± 6.83 × 10^1 a^
	2-Ethylfuran-D	3208-16-0	C_6_H_8_O	971.1	1.3158	1.26 × 10^2^ ± 3.75 × 10^1 d^	2.08 × 10^2^ ± 5.67 × 10^1 c^	5.56 × 10^2^ ± 3.57 × 10^1 a^	4.18 × 10^2^ ± 9.01 × 10^1 ab^	2.54 × 10^2^ ± 3.07 × 10^1 c^	2.83 × 10^2^ ± 6.36 × 10^1 bc^
	2-Ethylfuran-M	3208-16-0	C_6_H_8_O	976.3	1.04775	8.26 × 10^3^ ± 1.21 × 10^3 a^	1.07 × 10^3^ ± 2.23 × 10^2 b^	4.27 × 10^2^ ± 2.45 × 10^1 c^	4.48 × 10^2^ ± 4.59 × 10^1 c^	4.43 × 10^2^ ± 3.11 × 10^1 c^	4.16 × 10^2^ ± 4.59 × 10^1 c^
	2,3-Dimethyl-5-ethylpyrazine	15707-34-3	C_8_H_12_N_2_	1,499.2	1.22599	2.08 × 10^2^ ± 2.66 × 10^1 b^	2.32 × 10^2^ ± 1.75 × 10^1 b^	6.36 × 10^2^ ± 1.52 × 10^2 a^	5.75 × 10^2^ ± 9.65 × 10^1 a^	6.25 × 10^2^ ± 5.96 × 10^1 a^	6.31 × 10^2^ ± 5.97 × 10^1 a^
Acids	Acetic acid-D	64-19-7	C_2_H_4_O_2_	1,445.2	1.15187	1.26 × 10^2^ ± 2.03 × 10^1 c^	4.57 × 10^2^ ± 6.17 × 10^1 b^	6.30 × 10^2^ ± 7.15 × 10^1 a^	6.32 × 10^2^ ± 1.16 × 10^2 a^	5.55 × 10^2^ ± 2.80 × 10^1 ab^	6.05 × 10^2^ ± 9.32 × 10^1 ab^
	Acetic acid-M	64-19-7	C_2_H_4_O_2_	1,443.6	1.05353	3.94 × 10^3^ ± 1.21 × 10^2 c^	6.30 × 10^3^ ± 1.63 × 10^2 b^	6.92 × 10^3^ ± 3.30 × 10^2 a^	6.58 × 10^3^ ± 9.46 × 10^1 ab^	6.95 × 10^3^ ± 1.38 × 10^2 a^	6.73 × 10^3^ ± 1.29 × 10^2 a^
Others	Decalin	91-17-8	C_10_H_18_	1,156	1.34461	7.15 × 10^1^ ± 1.63 × 10^1 b^	6.71 × 10^1^ ± 9.91 ^b^	1.75 × 10^2^ ± 1.51 × 10^1 a^	1.50 × 10^2^ ± 3.01 × 10^1 a^	1.45 × 10^2^ ± 1.46 × 10^1 a^	1.44 × 10^2^ ± 2.39 × 10^1 a^
	P-cymene	99-87-6	C_10_H_14_	1,249.9	1.32991	1.32 × 10^2^ ± 1.72 × 10^1 c^	1.29 × 10^3^ ± 2.29 × 10^2 b^	2.04 × 10^3^ ± 1.83 × 10^2 a^	1.50 × 10^3^ ± 2.44 × 10^2 b^	1.40 × 10^3^ ± 1.34 × 10^2 b^	1.30 × 10^3^ ± 2.44 × 10^2 b^
	Dimethyl disulfide	624-92-0	C_2_H_6_S_2_	1,066.6	1.13349	2.01 × 10^2^ ± 3.72 × 10^1 c^	1.92 × 10^3^ ± 3.90 × 10^2 b^	3.46 × 10^3^ ± 4.07 × 10^2 a^	2.83 × 10^3^ ± 5.95 × 10^2 a^	1.67 × 10^3^ ± 2.72 × 10^2 b^	1.50 × 10^3^ ± 4.27 × 10^2 b^
	Undecane	1120-21-4	C_11_H_24_	1,111	1.08945	8.54 × 10^2^ ± 2.34 × 10^2 a^	5.65 × 10^2^ ± 6.09 × 10^1 ab^	5.14 × 10^2^ ± 3.70 × 10^1 b^	5.16 × 10^2^ ± 4.45 × 10^1 b^	6.64 × 10^2^ ± 5.44 × 10^1 ab^	6.75 × 10^2^ ± 6.21 × 10^1 ab^

^*^For each volatile compound, the same superscript after the sd value indicates a significant difference (*p* < 0.05).

^#^RI, RT and Dt represent retention index, retention time and drift time, respectively.

#### 3.2.2 Types of volatile compounds in SBD

**Figure 5:**
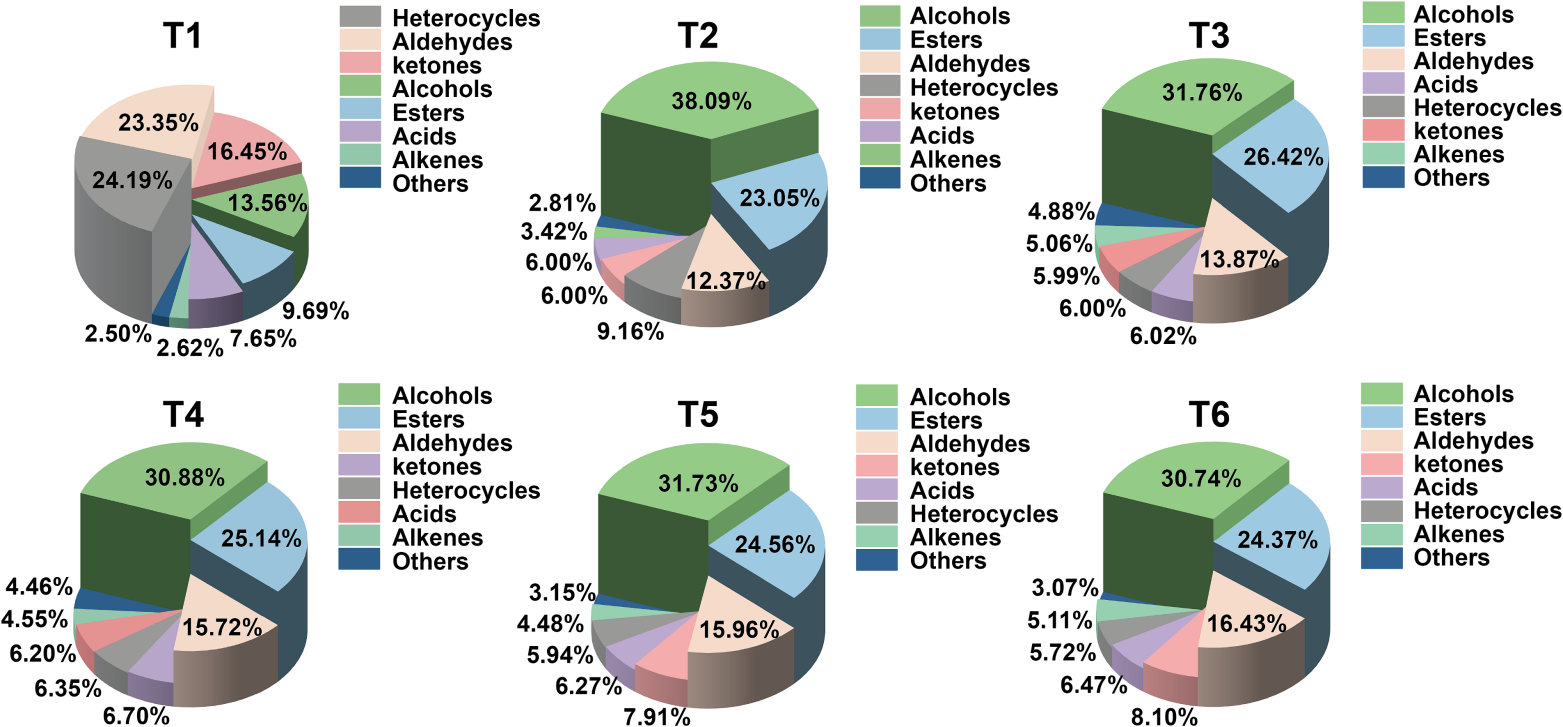
3D histogram based on the peak intensity of VOCs detected by GC-IMS in the T1–T6 processing stage of the SBD model.

In order to clearly show the differences of VOCs categories in the process of SBD, the peak intensity of the qualitative compounds was normalized to obtain a 3D histogram of relative proportion (Figure 5). The SBD VOCs consist of alcohols, esters, aldehydes, heterocyclics, ketones, acids, alkenes and other compounds. In the process of SBD processing, the VOCs with a large change in peak intensity are alcohols, esters and aldehydes. The peak of alcohols in the marinating stage (T2) was 39.09%, significantly higher than that in the control group (T1) (13.56%). However, the content of alcohols was only 30.74% at the end of steaming (T6). Compared with the control group, the peak intensity of esters in the marinating stage (T2) increased to 23.05%, and the peak intensity in the seasoning stage (T3) reached the highest, which was 26.42%, but in the subsequent steaming, it gradually decreased to 24.37%. The content of aldehydes gradually increased from the marinating stage (T2), and reached the highest value of 16.43% at the end of steaming.

### 3.3 ROAV calculations for volatile compounds in SBD

**Figure 6:**
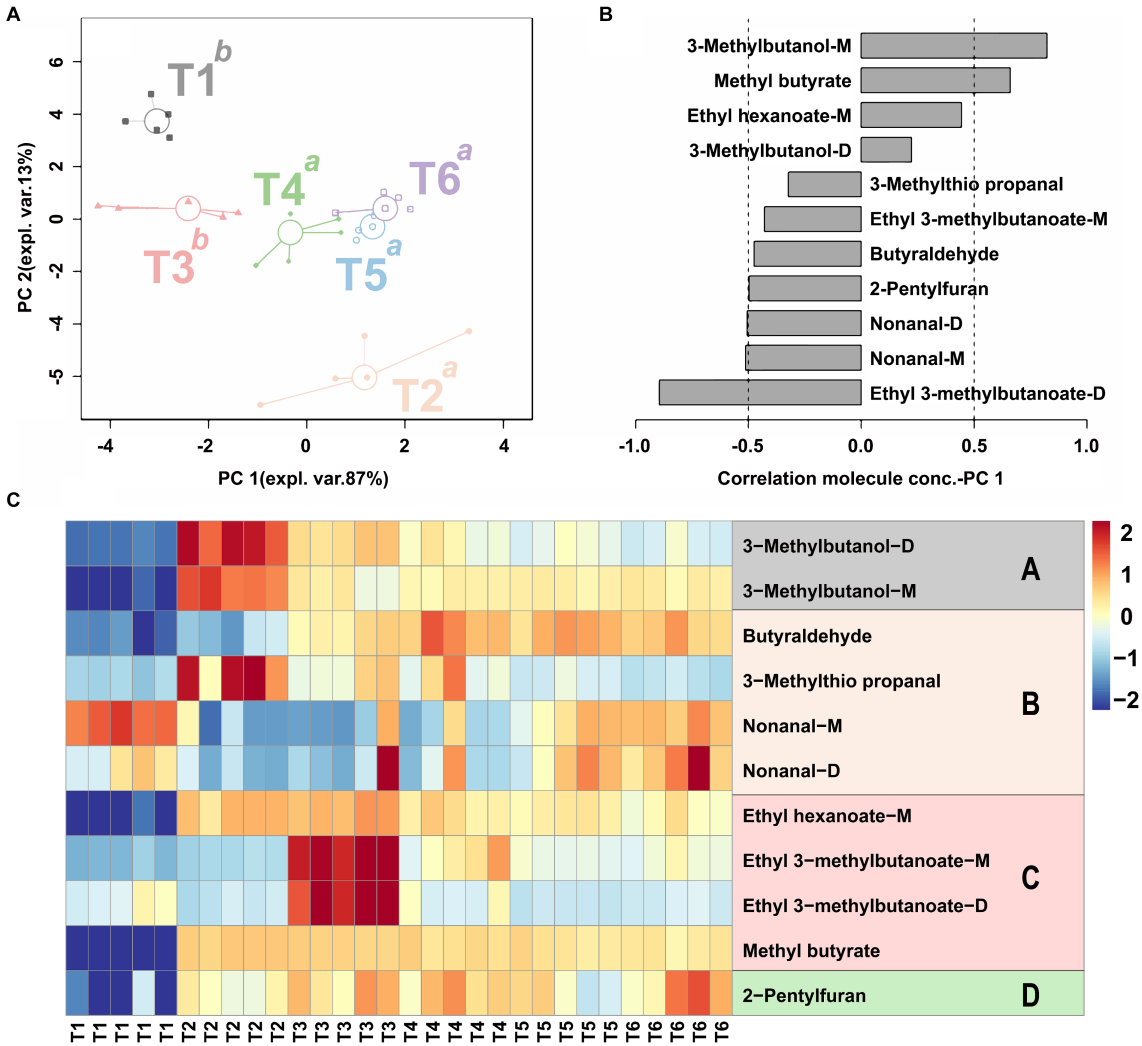
The rPCA model calculated based on the peak intensity has relative odor activity aroma compounds show significant differences in the peak intensity. The score plot **(A)** shows the overall trend of the sample in six stages of SBD. Superscript lowercase letters indicate significant differences between samples on PC 1. The loading plot **(B)** evidences significant correlations (*p* < 0.05) between the peak intensity of each VOCs and its importance over PC 1. The heat maps **(C)** depict aroma compounds’ relative odor activity in the SBD model at different stages, with letters A–D representing alcohols, aldehydes, esters, and heterocycles, respectively.

In this study, a total of 11 key aroma compounds, were successfully identified (Table 2) including 3-Methylbutanol-D, 3-Methylbutanol-M, Butyraldehyde, 3-Methylthio propanal, Nonanal-M, Nonanal-D, Ethyl hexanoate-M, Ethyl 3-methylbutanoate-M, Ethyl 3-methylbutanoate-D, Methyl butyrate and 2-Pentylfuran were successfully identified. To highlight the overall trend of VOCs above, an rPCA model and class heat map were built based on their peak intensity, as shown in Figure 6. In the rPCA model, PC1 accounted for 87% of the variation across the sample set, summarizing the differences between the groups (Figure 6A). In the SBD, the VOCs of the control group (T1) and steam for 60 min (T6) had the most significant difference. The content of 3-Methylbutanol-M, Methyl butyrate, Ethyl hexanoate-M and 3-Methylbutanol-D in T1 are the highest. In contrast, 3-Methylthio propanal, Ethyl 3-methyl butanoate-M, Butyraldehyde, 2-Pentylfuran, Nonanal-D, Nonanal-M and Ethyl 3-methyl butanoate-D were detected in T6 group (Figure 6B). Heat map analysis further shows that the peak intensity of esters of compounds with key aroma compounds in SBD samples was the highest, followed by alcohols and aldehydes (Figure 6C). Moreover, the peak intensity of aldehyde compounds Nonanal-M and Nonanal-D in raw meat (T1) was higher, the peak intensity of alcohol compounds 3-Methylbutanol-D, 3-Methylbutanol-M and aldehyde compound 3-Methylthio propanal were relatively higher in marinating stage (T2), and the peak intensity of ester compounds Ethyl 3-methylbutanoate-M, Ethyl 3-methyl butanoate-D were the highest in seasoning stage (T3). Additionally, as the cooking process progressed, the level of Butyraldehyde, Nonanal-M, Nonanal-D, 2-Pentylfuran showed an increasing trend, with the peak intensity of these compounds being highest at the end (T6) of the steaming process.

**Table 2:** The relative odor activity values of T1–T6 samples in SBD models.

Compounds	CAS	Threshold (μg/kg)	ROAV						Aroma description^*^
			T1	T2	T3	T4	T5	T6	
3-Methylbutanol-D^#^	123-51-3	6.10	11.67	15.93	12.90	18.86	28.03	23.31	Alcohol, bananas
3-Methylbutanol-M	123-51-3	6.10	2.21	4.35	4.15	7.54	11.85	11.79	Alcohol, bananas
Nonanal-M	124-19-6	3.10	15.51	1.46	1.35	3.05	7.94	8.22	Citrus, cucumber
2-Pentylfuran	3777-69-3	5.80	1.74	0.45	0.62	1.16	1.68	1.87	Butter, flowers
3-Methylthio propanal	3268-49-3	0.06	100.00	100.00	41.89	73.45	95.00	68.94	Cooked potatoes
Ethyl hexanoate-M	123-66-0	20.00	0.73	1.96	2.50	3.95	6.38	5.37	Fennel, apple peel
Ethyl 3-methylbutanoate-M	108-64-5	0.07	37.49	14.54	100.00	100.00	100.00	100.00	Fennel, apple
Ethyl 3-methylbutanoate-D	108-64-5	0.07	51.95	6.77	38.52	28.60	26.48	29.24	Fennel, apple
Nonanal-D	124-19-6	3.10	2.06	0.28	0.32	0.64	1.61	1.72	Citrus, cucumber
Methyl butyrate	623-42-7	59.00	0.30	0.53	0.67	1.24	1.83	1.77	Apple, banana
Butyraldehyde	123-72-8	100.00	2.29	0.41	0.65	1.33	2.13	1.95	Banana, pungent

^#^M represents a monomer, D represents a dimer.

^*^Volatile Substance description from Vcf-online system (Accessed on 18 March 2024 at 22:00, www.vcf-online.nl/VcfHome.cfm).

### 3.4 Analysis of free amino acids in taste (FAAs) of SBD

**Figure 7:**
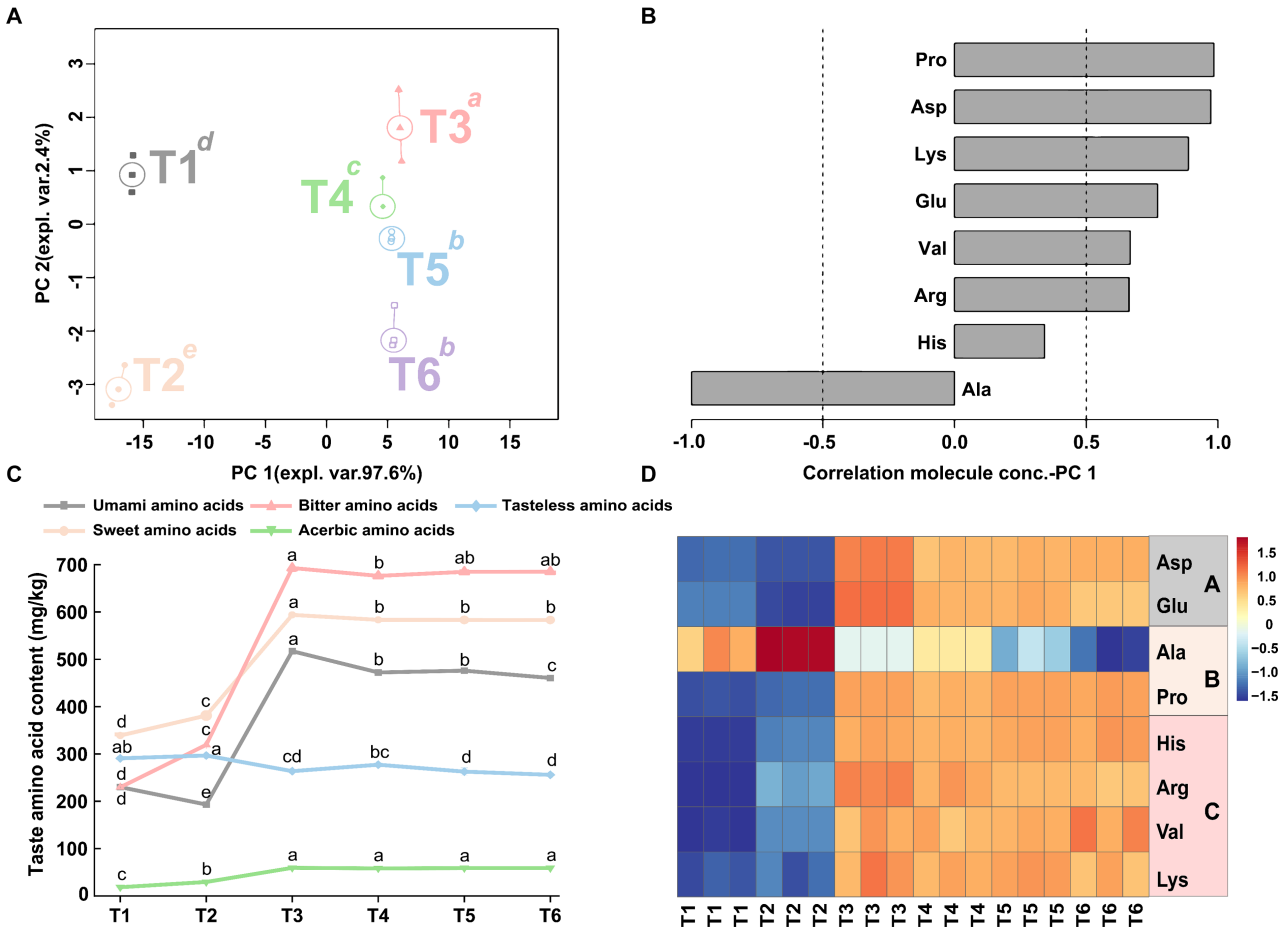
The rPCA model was established based on the content of amino acids with taste characteristics in SBD samples. The score plot **(A)** illustrates the overall trend of the samples. Lowercase superscript letters indicate significant differences (*p* < 0.05) between samples on PC1. Each sample group is depicted by a circle indicating its median value. The load plots **(B)** showcase the correlation between the taste activity value of each free amino acid and their significance on PC 1. The line graph **(C)** shows the variation trend of taste of T1–T6. The heat map **(D)** shows the ingredients with taste modifications in taste activity value of T1–T6, with the letters A–C representing umami, sweet, and bitter amino acids, respectively.

Free amino acids are an essential part that often contributes to taste. In this study, 19 important free amino acids in the SBD at different processing stages were characterized, and their TAV were further calculated to evaluate their contribution to the overall taste characteristics of the SBD. In all stages of SBD (T1–T6), only Glu has a TAV value greater than 1 in T3–T6, indicating that Glu has a core contribution to the overall taste after SBD marinating. The TAV values for other free amino acids were between 0.1 and 1, indicating that they contributed to SBD overall taste (Supplementary Table S1). In order to show the differences and overall trends of free amino acids in each stage of the SBD, an rPCA model and a class heat map were established according to the content of free amino acids, as shown in Figure 7. In the entire rPCA model, PC1 accounts for 97.6% of the total samples and explains most of the difference. Among them, the differences were most significant in the marinating stage (T2) and the seasoning stage (T3) (*p* < 0.05). In detail, the content of Ala was higher in the T2 stage, while Pro, Asp, Lys, Glu, Val, Arg, and His were higher in the T3 stage (Figures 7A,B).

Moreover, the free amino acids were further divided into umami, sweet, bitter, acerbic and tasteless amino acids for analysis. The results shown in Figure 7C showed that the umami, sweet and bitter amino acids significantly increased during the T2–T3 stages of the SBD production (*p* < 0.05). The content of tasteless amino acid changed little in each stage, while the content of acerbic amino acid remained at a low level all the time. The results of the heat map of different stages of SBD showed that the content of sweet amino acids Ala was higher in the T1 and the T2 stages (Figure 7D). In the seasoning stage (T3), the contents of umami amino acids Asp and Glu, sweet amino acid Pro and bitter amino acids His, Arg, Val, and Lys are higher. As the steaming process progresses, during the steaming (T4–T6) stages, the content of some amino acids begins to rise and gradually increases to the highest level at the end of the steaming process. Including Asp, Pro, His, and Val taste amino acids.

## 4 Discussion

The flavor profile of SBD is extremely complex. Therefore, combining different techniques should be considered as necessary to obtain SBD’s characteristic flavor profiles. In recent years, the integration of diverse methodologies has gained extensive application in the field of culinary science. Wu et al. found that the combination of electronic nose and HS-GC-IMS could comprehensively obtain the flavor characteristics of beef cooked in tomato sour soup (8). Xu successfully used gas chromatography and an automated amino acid analyzer to evaluate the contribution of the processing stages to the nonvolatile components of roasted lamb (22). To our knowledge, this study represents the inaugural endeavor to thoroughly investigate the flavor attributes of SBD throughout its preparation process, employing an array of analytical tools including the E-nose, E-tongue, GC-IMS, and automated amino acid analyzer.

In this investigation, it was discovered that E-noses and E-tongues possess significant capabilities in discerning and differentiating the flavor profiles of SBD, with promising prospects for their future utilization in real-time industrial monitoring of SBD quality. However, the E-nose and E-tongues has some limitations, such as the failure to identify the composition of the aroma and taste (11, 14). To address this issue, we further adopted HS-GC-IMS and amino acid analyzer technology. The HS-GC-IMS is used for trace gas analysis, while amino acid analyzer is the most common method for detecting taste components of meat products (12, 32, 33).

A total of 66 volatiles and 19 free amino acids, including alcohols, esters, aldehydes, heterocycles, ketones, acids and alkenes compounds, were successfully identified during the of processing SBD. To further clarify the contribution of aroma compounds to the odor activity of SBD, the characteristic aroma markers of SBD were screened by calculating ROAV value. A sum of 11 volatile compounds were obtained as characteristic markers (ROAV > 1). 3-Methylbutanol-D, 3-Methylbutanol-M, and 3-Methylthio propanal are the main aroma contributors during the marinating stage. Ethyl 3-methyl butanoate-M and Ethyl 3-methyl butanoate-D are the main aroma contributors in the seasoning stage. However, it is worthy to note that the ROAV values of 11 characteristic aroma marker compounds are the highest when the steaming time is 40 min (T5), indicating that the steaming further promotes the formation of SBD aroma. This is due to thermal degradation and the Maillard reaction that occur during the steaming process (34). This is also consistent with the research results of Gruffat which showed that the thermal degradation of lipids and Maillard reaction further promote the formation of meat aroma (35).

Alcohols mainly from polyunsaturated fatty acids and lipids degradation, which help to form the ideal aroma. Although alcohols exert a less pronounced influence on meat aroma compared to aldehydes, they play an important role in the overall aroma formation of SBD. The alcohols substance found in this investigation was 3-Methylbutanol. It rises gradually during the steaming process and reaches its highest level at the end of the steaming. Which provides the alcohol and bananas flavor for SBD and was also found in beef aged by Yu et al. (36). During the steaming process, the key factor in producing flavor was by promoting thermal oxidation reactions (37, 38). This can also partially explain why the contribution of 3-Methylbutanol aroma to SBD increases with the prolonged steaming time. In addition, the formation pathways of characteristic flavor of meat also include Maillard reaction and Strecker degradation reaction, lipid and Maillard interaction, including esters and aldehydes (39–41).

Esters, such as Ethyl hexanoate, Ethyl 3-methylbutanoate, and Methyl butyrate, are commonly present in fermented foods like fermented soybeans and jams (2, 42). These esters have a low odor threshold which enables them to contribute to the distinctive aroma of fennel and fruity in SBD (43). Some of the Ethyl 3-methylbutanoate in SBD may come from the flavoring added during the seasoning stage, which may contribute to a superior fruity flavor and fennel aroma.

Aldehydes are secondary by-products of lipid oxidation and are important volatiles in all meat products, affected by processing techniques, temperature and duration (18, 44). In this study, the aldehyde compounds of SBD were mainly formed during the steaming process. Although they have a low threshold, they play a crucial role in the overall odor profile of SBD. As the stewing time increases, the trend of aldehyde compounds increasing gradually can be observed, and the aromatic aldehydes observed during this process are mainly straight-chain aldehydes, like Nonanal and Butyraldehyde. These aldehydes have citrus, cucumber, banana, and spicy odor characteristics resulting from the oxidative thermal degradation of linoleic acid (45). 3-Methylthio propanal, a branched aldehyde obtained by the Sterck degradation of branched amino acids, will provide the characteristic aroma of cooked potato for SBD (46).

In terms of free amino acid analysis, there were significant differences in the 19 free amino acids of SBD, and the total amino acid content was the highest in the seasoning stage (Supplementary Table S1). Further calculation of taste activity value showed that the TAV value of a taste compound Glu was greater than 1, indicating that the taste of SBD can be detected online by labeling Glu, while Asp, Ala, His, Arg, Val, Lys can be used as potential taste markers for online detection of SBD taste. Glu serves as the hallmark amino acid for umami, the fifth primary taste sensation alongside sweet, sour, salty, and bitter (47). This distinctive taste perception primarily arises from the recognition of protein-coupled receptors specific to glutamate, such as mGluR4 and the heteromeric T1R1 + T1R3 receptor (48), while the combination of Ala and Glu can enhance the freshness of SBD, which is consistent with Jiang et al.’s research results (28). In addition, the study also found that the content of umami, sweet and bitter amino acids was the highest in the seasoning stage. As the steaming time increases, the flavor amino acids showed varying degrees of reduction, which may be due to the heat treatment under high heat conditions through the Maillard reaction to convert into small molecule aroma compounds including aldehydes, ketones, esters and alcohols (32, 49).

## 5 Conclusion

In this study, the E-nose and E-tongue in intelligent sensory technology were combined with an amino acid analyzer and HS-GC-IMS to track the aroma and taste changes of the SBD throughout the manufacturing process. The results showed that the intelligent sensory system can effectively recognize SBD’s aroma and taste characteristics. By HS-GC-IMS, 66 volatile organic compounds (VOCs) were successfully identified and further using the results of relative odor activity value (ROAV) assessment, it was shown that nine of these VOCs were essential for aroma contribution, namely. 3-Methylbutanol, 2-Pentylfuran, 3-Methylthio propanal, Ethyl hexanoate, Ethyl 3-methylbutanoate, Nonanal, Methyl butyrate and Butyraldehyde. Nineteen free amino acids were detected, and the subsequent taste activity value (TAV) revealed that Glu significantly contributed to the umami taste of SBD. Additionally, it was found that the Seasoning stage (T3) and Steaming (T5) played a crucial role in developing the SBD flavor. This study analyses the aroma and taste dynamics during the production of SBD to provide theoretical guidance and insights into the quality qualities of SBD during industrial processing to a certain extent.

## Data Availability

The datasets presented in this study can be found in online repositories. The names of the repository/repositories and accession number(s) can be found in the article/Supplementary material.

## Footnotes

^1^ https://www.omicstudio.cn

## References

[1] Ahn H-J Kim J-H Jo C Lee J-W Yook H-S Byun M-W. Effects of gamma irradiation on residual nitrite, residual ascorbate, color, and N-nitrosamines of cooked sausage during storage. Food Control. (2004) 15:197–203. doi: 10.1016/S0956-7135(03)00047-1

[2] Xia A-N Tang X-J Dong G-Z Lei S-M Liu Y-G Tian X-M. Quality assessment of fermented rose jams based on physicochemical properties, HS-GC-MS and HS-GC-IMS. LWT. (2021) 151:112153. doi: 10.1016/j.lwt.2021.112153

[3] Wang Z Cai R Yang X Gao Z Yuan Y Yue T. Changes in aroma components and potential Maillard reaction products during the stir-frying of pork slices. Food Control. (2021) 123:107855. doi: 10.1016/j.foodcont.2020.107855

[4] Bai S Wang Y Luo R Shen F Bai H Ding D. Formation of flavor volatile compounds at different processing stages of household stir-frying mutton sao zi in the northwest of China. LWT. (2021) 139:110735. doi: 10.1016/j.lwt.2020.110735

[5] Li W Zheng L Xiao Y Li L Wang N Che Z. Insight into the aroma dynamics of Dongpo pork dish throughout the production process using electronic nose and GC×GC-MS. LWT. (2022) 169:113970. doi: 10.1016/j.lwt.2022.113970

[6] Chen J Cai X Liu J Yuan C Yi Y Qiao M. Investigation of different ingredients affected the flavor changes of Yu-Shiang shredded pork by using GC-IMS and GC-MS combined with E-nose and E-tongue. Heliyon. (2024) 10:e31486. doi: 10.1016/j.heliyon.2024.e31486, PMID: 38828359 PMC11140597

[7] Zhu C Yang Z Lu X Yi Y Tian Q Deng J. Effects of *Saccharomyces cerevisiae* strains on the metabolomic profiles of Guangan honey pear cider. LWT. (2023) 182:114816. doi: 10.1016/j.lwt.2023.114816

[8] Wu W Wang X Hu P Zhang Y Li J Jiang J. Research on flavor characteristics of beef cooked in tomato sour soup by gas chromatography-ion mobility spectrometry and electronic nose. LWT. (2023) 179:114646. doi: 10.1016/j.lwt.2023.114646

[9] Nie S Li L Wang Y Wu Y Li C Chen S. Discrimination and characterization of volatile organic compound fingerprints during sea bass (Lateolabrax japonicas) fermentation by combining GC-IMS and GC-MS. Food Biosci. (2022) 50:102048. doi: 10.1016/j.fbio.2022.102048

[10] Lu W Chen J Li X Qi Y Jiang R. Flavor components detection and discrimination of isomers in Huaguo tea using headspace-gas chromatography-ion mobility spectrometry and multivariate statistical analysis. Anal Chim Acta. (2023) 1243:340842. doi: 10.1016/j.aca.2023.340842, PMID: 36697178

[11] Zhang Q Ma J Yang Y Deng J Zhu K Yi Y. Effects of *S. cerevisiae* strains on the sensory characteristics and flavor profile of kiwi wine based on E-tongue, GC-IMS and 1H-NMR. LWT. (2023) 185:115193. doi: 10.1016/j.lwt.2023.115193

[12] Wang S Chen H Sun B. Recent progress in food flavor analysis using gas chromatography–ion mobility spectrometry (GC–IMS). Food Chem. (2020) 315:126158. doi: 10.1016/j.foodchem.2019.126158, PMID: 32014672

[13] Lin R Yuan H Wang C Yang Q Guo Z. Study on the flavor compounds of Fo Tiao Qiang under different thawing methods based on GC–IMS and electronic tongue technology. Food Secur. (2022) 11:1330. doi: 10.3390/foods11091330, PMID: 35564052 PMC9099569

[14] Lu L Hu Z Hu X Li D Tian S. Electronic tongue and electronic nose for food quality and safety. Food Res Int. (2022) 162:112214. doi: 10.1016/j.foodres.2022.112214, PMID: 36461383

[15] Yao W Cai Y Liu D Chen Y Li J Zhang M. Analysis of flavor formation during production of Dezhou braised chicken using headspace-gas chromatography-ion mobility spectrometry (HS-GC-IMS). Food Chem. (2022) 370:130989. doi: 10.1016/j.foodchem.2021.130989, PMID: 34509944

[16] Xiong Y Guan J Wu B Wang T Yi Y Tang W. Exploring the profile contributions in Meyerozyma guilliermondii YB4 under different NaCl concentrations using GC-MS combined with GC-IMS and an electronic nose. Molecules. (2023) 28:6979. doi: 10.3390/molecules28196979, PMID: 37836821 PMC10574234

[17] Wu B Zhu C Deng J Dong P Xiong Y Wu H. Effect of Sichuan pepper (zanthoxylum genus) addition on flavor profile in fermented ciba chili (capsicum genus) using GC-IMS combined with E-nose and E-tongue. Molecules. (2023) 28:5884. doi: 10.3390/molecules28155884, PMID: 37570854 PMC10420873

[18] Chen J Tao L Zhang T Zhang J Wu T Luan D. Effect of four types of thermal processing methods on the aroma profiles of acidity regulator-treated tilapia muscles using E-nose, HS-SPME-GC-MS, and HS-GC-IMS. LWT. (2021) 147:111585. doi: 10.1016/j.lwt.2021.111585

[19] Wang Y Wang D Lv Z Zeng Q Fu X Chen Q. Analysis of the volatile profiles of kiwifruits experiencing soft rot using E-nose and HS-SPME/GC–MS. LWT. (2023) 173:114405. doi: 10.1016/j.lwt.2022.114405

[20] Deng Y Wang R Zhang Y Li X Gooneratne R Li J. Comparative analysis of flavor, taste, and volatile organic compounds in opossum shrimp paste during long-term natural fermentation using E-nose, E-tongue, and HS-SPME-GC-MS. Food Secur. (2022) 11:1938. doi: 10.3390/foods11131938, PMID: 35804754 PMC9266136

[21] Liu N Shen S Huang L Deng G Wei Y Ning J. Revelation of volatile contributions in green teas with different aroma types by GC–MS and GC–IMS. Food Res Int. (2023) 169:112845. doi: 10.1016/j.foodres.2023.112845 37254419

[22] Xu Y Zhang D Liu H Wang Z Hui T Sun J. Comprehensive evaluation of volatile and nonvolatile compounds in oyster cuts of roasted lamb at different processing stages using traditional Nang roasting. Food Secur. (2021) 10:1508. doi: 10.3390/foods10071508, PMID: 34210029 PMC8306727

[23] Cai X Zhu K Li W Peng Y Yi Y Qiao M. Characterization of flavor and taste profile of different radish (*Raphanus Sativus* L.) varieties by headspace-gas chromatography-ion mobility spectrometry (GC/IMS) and E-nose/tongue. Food Chem X. (2024) 22:101419. doi: 10.1016/j.fochx.2024.101419 38756475 PMC11096940

[24] Lu Y Wang Y Zhao G Yao Y. Identification of aroma compounds in Zhuhoujiang, a fermented soybean paste in Guangdong China. LWT. (2021) 142:111057. doi: 10.1016/j.lwt.2021.111057

[25] Xu J Zhang Y Yan F Tang Y Yu B Chen B. Monitoring changes in the volatile compounds of tea made from summer tea leaves by GC-IMS and HS-SPME-GC-MS. Food Secur. (2022) 12:146. doi: 10.3390/foods12010146, PMID: 36613362 PMC9818854

[26] Gao L Zhang L Liu J Zhang X Lu Y. Analysis of the volatile flavor compounds of pomegranate seeds at different processing temperatures by GC-IMS. Molecules. (2023) 28:2717. doi: 10.3390/molecules28062717, PMID: 36985689 PMC10052118

[27] Fang X Xu W Jiang G Sui M Xiao J Ning Y. Monitoring the dynamic changes in aroma during the whole processing of Qingzhuan tea at an industrial scale: from fresh leaves to finished tea. Food Chem. (2024) 439:137810. doi: 10.1016/j.foodchem.2023.137810, PMID: 38043275

[28] Jiang S Zhu Y Peng J Zhang Y Zhang W Liu Y. Characterization of stewed beef by sensory evaluation and multiple intelligent sensory technologies combined with chemometrics methods. Food Chem. (2023) 408:135193. doi: 10.1016/j.foodchem.2022.135193, PMID: 36563617

[29] Box GEP Cox DR. An analysis of transformations. J Roy Stat Soc Ser B. (2018) 26:211–43. doi: 10.1111/j.2517-6161.1964.tb00553.x

[30] Zhou B Liu X Lan Q Wan F Yang Z Nie X. Comparison of aroma and taste profiles of kiwi wine fermented with/without peel by combining intelligent sensory, gas chromatography-mass spectrometry, and proton nuclear magnetic resonance. Food Secur. (2024) 13:1729. doi: 10.3390/foods13111729, PMID: 38890957 PMC11172059

[31] Hubert M Rousseeuw PJ. ROBPCA: a new approach to robust principal component analysis. Technometrics. (2005) 47:64–79. doi: 10.1198/004017004000000563

[32] Chu Y Ding Z Wang J Xie J. Exploration of the evolution and production of volatile compounds in grouper (*Epinephelus coioides*) during cold storage. Food Biosci. (2023) 52:102496. doi: 10.1016/j.fbio.2023.102496

[33] Zhang Z Sun H Liu J Zhang H Huang F. Changes in eating quality of Chinese braised beef produced from three different muscles. Int J Gastron Food Sci. (2022) 29:100584. doi: 10.1016/j.ijgfs.2022.100584

[34] Zhang M Chen M Fang F Fu C Xing S Qian C. Effect of sous vide cooking treatment on the quality, structural properties and flavor profile of duck meat. Int J Gastron Food Sci. (2022) 29:100565. doi: 10.1016/j.ijgfs.2022.100565

[35] Gruffat D Bauchart D Thomas A Parafita E Durand D. Fatty acid composition and oxidation in beef muscles as affected by ageing times and cooking methods. Food Chem. (2021) 343:128476. doi: 10.1016/j.foodchem.2020.128476 33158683

[36] Yu H Zhang S Liu X Lei Y Wei M Liu Y. Comparison of physiochemical attributes, microbial community, and flavor profile of beef aged at different temperatures. Front Microbiol. (2022) 13:1091486. doi: 10.3389/fmicb.2022.1091486, PMID: 36620023 PMC9813384

[37] Wojtasik-Kalinowska I Szpicer A Binkowska W Hanula M Marcinkowska-Lesiak M Poltorak A. Effect of processing on volatile organic compounds formation of meat—review. Appl Sci. (2023) 13:705. doi: 10.3390/app13020705

[38] Li J Han D Huang F Zhang C. Effect of reheating methods on eating quality, oxidation and flavor characteristics of braised beef with potatoes dish. Int J Gastron Food Sci. (2023) 31:100659. doi: 10.1016/j.ijgfs.2023.100659

[39] Rao J Meng F Li Y Chen W Liu D Zhang J. Effect of cooking methods on the edible, nutritive qualities and volatile flavor compounds of rabbit meat. J Sci Food Agric. (2022) 102:4218–28. doi: 10.1002/jsfa.11773, PMID: 35038172

[40] Cheng L Li X Tian Y Wang Q Li X An F. Mechanisms of cooking methods on flavor formation of Tibetan pork. Food Chem X. (2023) 19:100873. doi: 10.1016/j.fochx.2023.100873, PMID: 37745033 PMC10511784

[41] Liu Z Huang Y Kong S Miao J Lai K. Selection and quantification of volatile indicators for quality deterioration of reheated pork based on simultaneously extracting volatiles and reheating precooked pork. Food Chem. (2023) 419:135962. doi: 10.1016/j.foodchem.2023.135962, PMID: 37004364

[42] Chen Y Li P Liao L Qin Y Jiang L Liu Y. Characteristic fingerprints and volatile flavor compound variations in Liuyang Douchi during fermentation via HS-GC-IMS and HS-SPME-GC-MS. Food Chem. (2021) 361:130055. doi: 10.1016/j.foodchem.2021.130055, PMID: 34023693

[43] Xie B Xu Y Yao Z Zhu B Li X Sun Y. Effects of different thermal treatment temperatures on volatile flavour compounds of water-boiled salted duck after packaging. LWT. (2022) 154:112625. doi: 10.1016/j.lwt.2021.112625

[44] Yang Y Zhang X Wang Y Pan D Sun Y Cao J. Study on the volatile compounds generated from lipid oxidation of Chinese bacon (unsmoked) during processing. Euro J Lipid Sci Tech. (2017) 119:1600512. doi: 10.1002/ejlt.201600512

[45] Zeng Z Zhang H Chen JY Zhang T Matsunaga R. Direct extraction of volatiles of Rice during cooking using solid-phase microextraction. Cereal Chem. (2007) 84:423–7. doi: 10.1094/CCHEM-84-5-0423

[46] Martínez-Onandi N Rivas-Cañedo A Nuñez M Picon A. Effect of chemical composition and high pressure processing on the volatile fraction of serrano dry-cured ham. Meat Sci. (2016) 111:130–8. doi: 10.1016/j.meatsci.2015.09.004, PMID: 26398007

[47] Kondoh T Torii K. MSG intake suppresses weight gain, fat deposition, and plasma leptin levels in male Sprague–Dawley rats. Physiol Behav. (2008) 95:135–44. doi: 10.1016/j.physbeh.2008.05.010 18559279

[48] Kong Y Yang X Ding Q Zhang Y-Y Sun B-G Chen H-T. Comparison of non-volatile umami components in chicken soup and chicken enzymatic hydrolysate. Food Res Int. (2017) 102:559–66. doi: 10.1016/j.foodres.2017.09.038, PMID: 29195986

[49] Khan MI Jo C Tariq MR. Meat flavor precursors and factors influencing flavor precursors—a systematic review. Meat Sci. (2015) 110:278–84. doi: 10.1016/j.meatsci.2015.08.002 26319308

