# Supplementary material for: Characterization of flavor profile of Steamed beef with rice flour using gas chromatography-ion mobility spectrometry combined with intelligent sensory (Electronic nose and tongue)
Source: Front Nutr. 2024 Aug 20;11:1435364. doi: 10.3389/fnut.2024.1435364 (PMC11368871; doi:10.3389/fnut.2024.1435364)
Supplement: Supplementary file 1 [file Table_1.docx]

Characterization of flavor profile of Steamed beef with rice flour using Gas Chromatography-Ion Mobility Spectrometry (GC-IMS) combined with intelligent sensory (Electronic nose and tongue)

# Supplementary Tables

**Table S1.** Content of free amino acids and taste activity of SBD at different processing stages (mean ± sd).

| Compounds | Thresholds mg/100g | Content (mg/kg) | | | | | | TAV | | | | | |
| --- | --- | --- | --- | --- | --- | --- | --- | --- | --- | --- | --- | --- | --- |
|  |  | T1 | T2 | T3 | T4 | T5 | T6 | T1 | T2 | T3 | T4 | T5 | T6 |
| Umami |  | | | | | | | | | | | | |
| Asp | 100 | 4.52×10^1^ ± 3.43×10^-1^ ^d #^ | 4.15×10^1^ ± 2.18×10^-1^ ^e^ | 1.14×10^2^ ± 6.78×10^-1^ ^a^ | 1.04×10^2^ ± 1.25 ^c^ | 1.05×10^2^ ± 9.03×10^-1^ ^bc^ | 1.07×10^2^ ± 2.00×10^-3^ ^b^ | 0.05 ± 0.01 | 0.04 ± 0.00 | 0.11 ± 0.00 | 0.10 ± 0.01 | 0.11 ± 0.01 | 0.11 ± 0.00 |
| Glu | 30 | 1.85×10^2^ ± 7.35×10^-2^ ^d^ | 1.52×10^2^ ± 1.76 ^e^ | 4.03×10^2^ ± 7.37×10^-1^ ^a^ | 3.68×10^2^ ± 1.22 ^b^ | 3.71×10^2^ ± 1.87 ^b^ | 3.54×10^2^ ± 1.17 ^c^ | 0.62 ± 0.00 | 0.51 ± 0.01 | 1.34 ± 0.01 | 1.23 ± 0.01 | 1.24 ± 0.01 | 1.18 ± 0.00 |
| Sweet |  | | | | | | | | | | | | |
| Ala | 60 | 2.15×10^2^ ± 1.26 ^b^ | 2.21×10^2^ ± 1.56×10^-1^ ^a^ | 2.10×10^2^ ± 1.50×10^-2^ ^d^ | 2.13×10^2^ ± 8.00×10^-3^ ^c^ | 2.07×10^2^ ± 1.41 ^e^ | 2.03×10^2^ ± 1.04 ^f^ | 0.36 ± 0.00 | 0.37 ± 0.00 | 0.35 ± 0.00 | 0.35 ± 0.00 | 0.35 ± 0.01 | 0.34 ± 0.00 |
| Thr | 260 | 1.92×10^1^ ± 6.80×10^-1^ ^c^ | 2.63×10^1^ ± 2.27×10^-1^ ^b^ | 6.90×10^1^ ± 2.60×10^-1^ ^a^ | 6.77×10^1^ ± 2.66×10^-1^ ^a^ | 6.77×10^1^ ± 2.19 ^a^ | 6.86×10^1^ ± 1.19 ^a^ | 0.01 ± 0.00 | 0.01 ± 0.00 | 0.03 ± 0.00 | 0.03 ± 0.00 | 0.03 ± 0.00 | 0.03 ± 0.00 |
| Ser | 150 | 2.68×10^1^ ± 6.35×10^-2^ ^e^ | 3.79×10^1^ ± 7.40×10^-2^ ^d^ | 1.12×10^2^ ± 1.47×10^-1^ ^a^ | 1.07×10^2^ ± 1.54×10^-1^ ^c^ | 1.10×10^2^ ± 1.40 ^b^ | 1.13×10^2^ ± 1.26×10^-1^ ^a^ | 0.02 ± 0.00 | 0.03 ± 0.00 | 0.07 ± 0.00 | 0.07 ± 0.00 | 0.07 ± 0.00 | 0.08 ± 0.00 |
| Gly | 130 | 4.57×10^1^ ± 9.00×10^-2^ ^e^ | 4.82×10^1^ ± 1.50×10^-2^ ^d^ | 6.62×10^1^ ± 7.56×10^-1^ ^a^ | 6.40×10^1^ ± 9.05×10^-2^ ^b^ | 6.27×10^1^ ± 3.73×10^-1^ ^c^ | 6.36×10^1^ ± 1.40×10^-1^ ^bc^ | 0.04 ± 0.00 | 0.04 ± 0.00 | 0.05 ± 0.00 | 0.05 ± 0.00 | 0.05 ± 0.00 | 0.05 ± 0.00 |
| Pro | 300 | 1.90×10^1^ ± 1.18×10^-1^ ^d^ | 2.47×10^1^ ± 7.26×10^-1^ ^c^ | 1.08×10^2^ ± 2.20×10^-2^ ^a^ | 1.05×10^2^ ± 1.50 ^b^ | 1.09×10^2^ ± 4.08×10^-1^ ^a^ | 1.08×10^2^ ± 1.24 ^a^ | 0.01 ± 0.00 | 0.01 ± 0.00 | 0.04 ± 0.00 | 0.03 ± 0.01 | 0.04 ± 0.00 | 0.04 ± 0.00 |
| Met | 30 | 1.29×10^1^ ± 6.46×10^-1^ ^d^ | 2.30×10^1^ ± 1.88×10^-1^ ^c^ | 2.88×10^1^ ± 9.86×10^-1^ ^a^ | 2.68×10^1^ ± 8.76×10^-1^ ^b^ | 2.65×10^1^ ± 2.03×10^-1^ ^b^ | 2.68×10^1^ ± 5.45×10^-1^ ^b^ | 0.04 ± 0.01 | 0.08 ± 0.00 | 0.10 ± 0.01 | 0.09 ± 0.00 | 0.09 ± 0.00 | 0.09 ± 0.00 |
| Bitter |  | | | | | | | | | | | | |
| His | 20 | 1.74×10^1^ ± 9.25×10^-2^ ^d^ | 2.09×10^1^ ± 5.35×10^-2^ ^c^ | 3.88×10^1^ ± 2.46×10^-1^ ^ab^ | 3.83×10^1^ ± 6.50×10^-2^ ^b^ | 3.89×10^1^ ± 2.36×10^-1^ ^ab^ | 3.92×10^1^ ± 5.64×10^-1^ ^a^ | 0.09 ± 0.00 | 0.10 ± 0.01 | 0.19 ± 0.01 | 0.19 ± 0.00 | 0.19 ± 0.01 | 0.20 ± 0.01 |
| Arg | 50 | 3.70×10^1^ ± 9.18×10^-1^ ^e^ | 6.70×10^1^ ± 2.41 ^d^ | 1.42×10^2^ ± 1.18 ^a^ | 1.35×10^2^ ± 3.49 ^b^ | 1.31×10^2^ ± 3.74×10^-1^ ^bc^ | 1.28×10^2^ ± 1.90 ^c^ | 0.07 ± 0.01 | 0.13 ± 0.01 | 0.28 ± 0.01 | 0.27 ± 0.01 | 0.26 ± 0.00 | 0.26 ± 0.01 |
| Val | 40 | 3.60×10^1^ ± 4.66×10^-1^ ^c^ | 4.78×10^1^ ± 7.00×10^-2^ ^b^ | 9.54×10^1^ ± 3.07 ^a^ | 9.42×10^1^ ± 2.79 ^a^ | 9.55×10^1^ ± 9.36×10^-1^ ^a^ | 1.00×10^2^ ± 4.14 ^a^ | 0.09 ± 0.00 | 0.12 ± 0.00 | 0.24 ± 0.01 | 0.24 ± 0.01 | 0.24 ± 0.00 | 0.25 ± 0.01 |
| Ile | 90 | 2.57×10^1^ ± 1.65×10^-1^ ^c^ | 3.59×10^1^ ± 4.67×10^-1^ ^b^ | 8.21×10^1^ ± 4.06×10^-1^ ^a^ | 8.15×10^1^ ± 2.80×10^-2^ ^a^ | 8.22×10^1^ ± 1.30 ^a^ | 8.27×10^1^ ± 3.09×10^-1^ ^a^ | 0.03 ± 0.00 | 0.04 ± 0.00 | 0.09 ± 0.00 | 0.09 ± 0.00 | 0.09 ± 0.00 | 0.09 ± 0.00 |
| Leu | 190 | 4.23×10^1^ ± 1.47×10^-1^ ^d^ | 6.34×10^1^ ± 5.90×10^-2^ ^c^ | 1.40×10^2^ ± 1.19×10^-1^ ^a^ | 1.37×10^2^ ± 2.32×10^-1^ ^b^ | 1.40×10^2^ ± 1.36 ^a^ | 1.39×10^2^ ± 6.27×10^-2^ ^a^ | 0.02 ± 0.00 | 0.03 ± 0.00 | 0.07 ± 0.00 | 0.07 ± 0.00 | 0.07 ± 0.00 | 0.07 ± 0.00 |
| Phe | 90 | 2.26×10^1^ ± 1.10 ^d^ | 3.20×10^1^ ± 1.08 ^c^ | 7.77×10^1^ ± 1.53×10^-1^ ^b^ | 7.76×10^1^ ± 2.23 ^b^ | 8.17×10^1^ ± 2.27 ^ab^ | 8.41×10^1^ ± 2.87 ^a^ | 0.03 ± 0.01 | 0.04 ± 0.01 | 0.09 ± 0.00 | 0.09 ± 0.01 | 0.09 ± 0.00 | 0.09 ± 0.01 |
| Lys | 50 | 4.86×10^1^ ± 1.55 ^b^ | 5.23×10^1^ ± 4.23 ^b^ | 1.17×10^2^ ± 5.55 ^a^ | 1.13×10^2^ ± 2.27 ^a^ | 1.16×10^2^ ± 6.61×10^-1^ ^a^ | 1.12×10^2^ ± 3.70 ^a^ | 0.10 ± 0.01 | 0.10 ± 0.01 | 0.23 ± 0.01 | 0.23 ± 0.01 | 0.23 ± 0.00 | 0.22 ± 0.01 |
| Acerbic |  | | | | | | | | | | | | |
| Tyr | - | 1.83×10^1^ ± 6.45×10^-2^ ^c^ | 2.94×10^1^ ± 1.31×10^-1^ ^b^ | 5.94×10^1^ ± 1.21×10^-1^ ^a^ | 5.82×10^1^ ± 3.85×10^-1^ ^a^ | 5.89×10^1^ ± 1.01 ^a^ | 5.87×10^1^ ± 2.90×10^-1^ ^a^ |  |  |  |  |  |  |
| Tasteless |  | | | | | | | | | | | | |
| Asn | - | 1.86×10^1^ ± 3.36×10^-1^ ^d^ | 3.15×10^1^ ± 7.65×10^-2^ ^c^ | 9.90×10^1^ ± 1.74×10^-1^ ^b^ | 1.01×10^2^ ± 6.22×10^-1^ ^b^ | 1.05×10^2^ ± 2.78 ^a^ | 9.92×10^1^ ± 1.80×10^-1^ ^b^ |  |  |  |  |  |  |
| GABA | - | 1.35 ± 4.00×10^-3^ ^f^ | 1.56 ± 3.50×10^-3^ ^e^ | 1.53×10^1^ ± 1.30×10^-2^ ^d^ | 1.55×10^1^ ± 1.50×10^-2^ ^c^ | 1.73×10^1^ ± 1.04×10^-1^ ^a^ | 1.63×10^1^ ± 1.25×10^-2^ ^b^ |  |  |  |  |  |  |
| Trp | - | 2.71×10^2^ ± 1.66 ^a^ | 2.64×10^2^ ± 3.64 ^a^ | 1.49×10^2^ ± 2.98 ^bc^ | 1.60×10^2^ ± 1.03 ^b^ | 1.40×10^2^ ± 1.10×101 ^c^ | 1.40×10^2^ ± 4.95×10^-1^ ^c^ |  |  |  |  |  |  |

# For each free amino acid, the same superscript after the sd value indicated a significant difference (p < 0.05).
